# Role of epigenetic in cancer biology, in hematologic malignancies and in anticancer therapy

**DOI:** 10.3389/fmmed.2024.1426454

**Published:** 2024-09-06

**Authors:** Armel Hervé Nwabo Kamdje, Hervet Paulain Dongmo Fogang, Patrice N. Mimche

**Affiliations:** ^1^ Department of Physiological Sciences and Biochemistry, Faculty of Medicine and Biomedical Sciences, University of Garoua, Garoua, Cameroon; ^2^ Department of Dermatology, Indiana University School of Medicine, Indianapolis, IN, United States; ^3^ Department of Medicine, Division of Gastroenterology and Hepatology, Indiana University School of Medicine, Indianapolis, IN, United States

**Keywords:** epigenetic processes, hematologic malignancy, risk factors, anticancer treatment, interindividual variability, chemoresistance

## Abstract

Major epigenetic changes are associated with carcinogenesis, including aberrant DNA methylations and post-translational modifications of histone. Indeed evidence accumulated in recent years indicates that inactivating DNA hypermethylation preferentially targets the subset of polycomb group (PcG) genes that are regulators of developmental processes. Conversely, activating DNA hypomethylation targets oncogenic signaling pathway genes, but outcomes of both events lead in the overexpression of oncogenic signaling pathways that contribute to the stem-like state of cancer cells. On the basis of recent evidence from population-basedclinical and experimental studies, we hypothesize that factors associated with risk for developing a hematologic malignancy (HM), such as metabolic syndrome and chronic inflammation, may trigger epigenetic mechanisms to increase the transcriptional expression of oncogenes and activate oncogenic signaling pathways. Signaling pathways associated with such risk factors include but are not limited to pro-inflammatory nuclear factor κB (NF-κB) and mitogenic, growth, and survival Janus kinase (JAK) intracellular non-receptor tyrosine kinase-triggered pathways. The latter includes signaling pathways such as transducer and activator of transcription (STAT), Ras GTPases/mitogen-activated protein kinases (MAPKs)/extracellular signal-related kinases (ERKs), phosphatidylinositol 3-kinase (PI3K)/Akt/mammalian target of rapamycin (mTOR), and β-catenin pathways. Recent findings on epigenetic mechanisms at work in the biology of cancer and in HMs and their importance in the etiology and pathogenesis of these diseases are herein summarized and discussed. Furthermore, the role of epigenetic processes in the determination of biological identity, the consequences for interindividual variability in disease clinical profile, and the potential of epigenetic drugs in HMs are also considered.

## 1 Introduction

A lot of investigations have shown that the epigenetic mechanisms are involved in regulation of all biological process in the body from conception to death. During an individual’s lifetime, epigenetic regulators integrate external factors, such as toxins, diet or drugs, and internal factors present in the cellular microenvironment like cytokines or growth factors (Wang T. P. et al., 2012), for gene expression regulation. This is achieved mainly by controlling genes to be selectively expressed or suppressed in the presence of specific signaling molecules through heritable changes in gene expression occurring without DNA sequence modification. Considering such role, it can be anticipated that epigenetic regulators are major players in the biological processes resulting in the establishment of personal molecular identity.

Aberrant epigenetic modifications can participate to the acquisition of considerable capabilities during tumor development and malignant progression. Not surprisingly, in hematologic malignancies (HMs), whose clinical features display a high inter-individual variability even for diseases originating from the same cell types, a large body of recent data suggests a pivotal pathogenic role for disease-associated epigenetic changes. HMs or blood cancers include cancers originating from adult primary hematopoietic organs (e.g., bone marrow, blood) such as myelomas and leukemias, and those originating from secondary lymphoid organs (e.g., lymph nodes) such as lymphomas. Unfortunately hematopoiesis and oncogenesis of hematologic malignancies are profoundly affected by epigenetic regulation. The occurrence of chemoresistance to classical anticancer drugs has raised the need for new therapeutic approaches. Furthermore, the incidence of HMs has been increasing (Liang and Turcan, 2022), at least in part due to the growing incidence of chronic diseases and conditions associated with major risk factors like metabolic syndrome and chronic inflammation. Epigenetic changes are reversible (Chandra, 2021), thus, constitute attractive pharmacological targets for HMs.

The present review critically summarizes recent evidence on risk factors and changes affecting the epigenetic regulation in HMs. The role of epigenetic processes in personal molecular identity determination, the consequences for interindividual variability in disease clinical profile, and the potential of epigenetic drugs in HMs will also be considered.

## 2 Normal epigenetic regulation and cancer epigenome

### 2.1 DNA methylation: transcriptional control of gene expression

DNA is packaged and structured into nucleosomes, basic units of the chromatin, by highly alkaline proteins named histones. Packaged genes are in nucleosomes in a transcriptionally repressed configuration, and are maintained inactive by DNA methylation (Hung et al., 2010) which is one of several epigenetic mechanisms that cells use to control gene expression.

**FIGURE 1:**
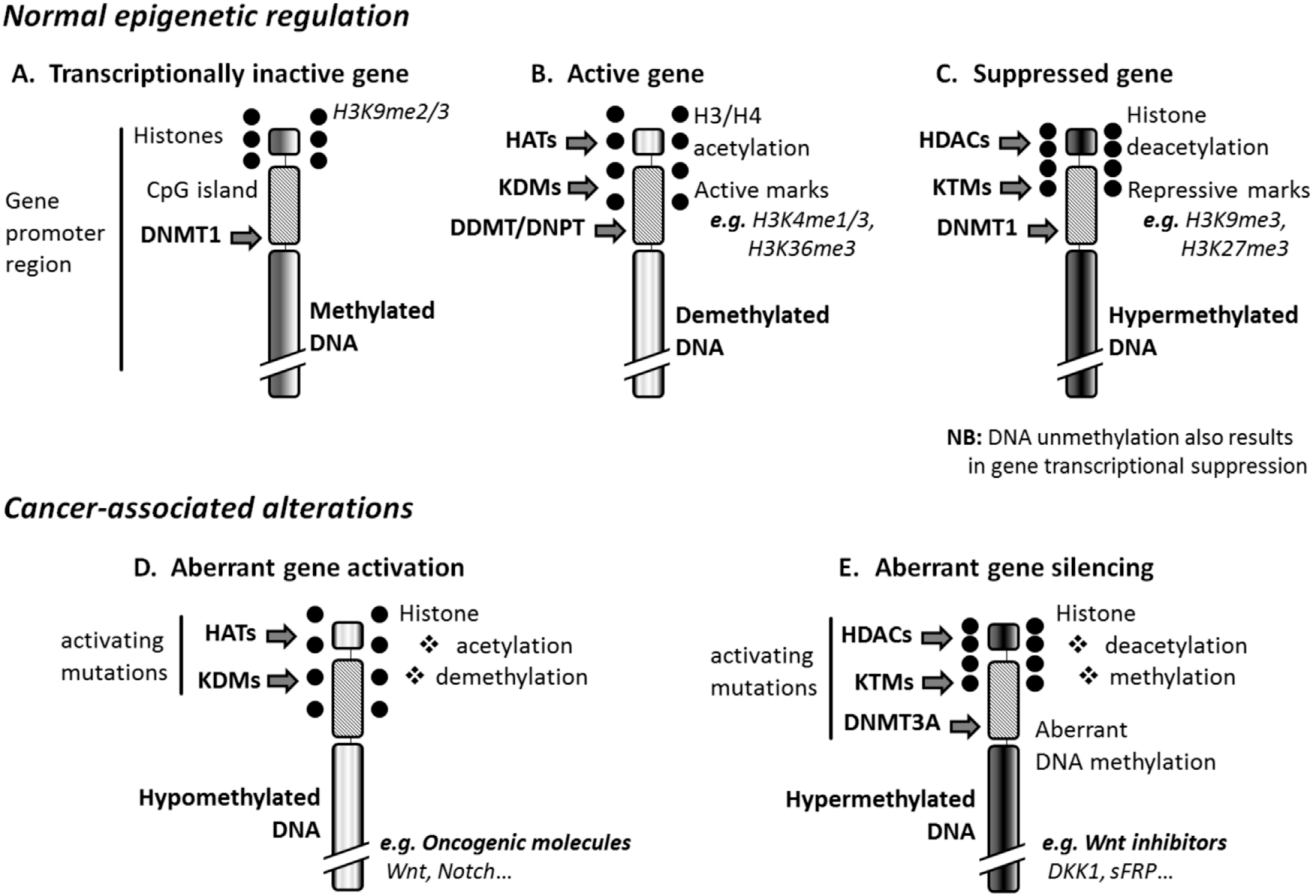
Epigenetic regulation and cancer. **(A)** Normal inactive genes are repressed, through DNA methylation, by DNA methyltransferase 1 (DNMT1), and express repressive marks such as histone 3 lysine 9 dimethylation (H3K9me2) or trimethylation (H3K9me3). **(B)** The activation of genes results from DNA-demethylation mediated by the enzymatic activity of DNA demethylases (DDMTs) and DNA glycosylases (DNPTs), and can be induced by histone demethylases (KDMs) and histone acetyltransferases (HATs). Such genes express the active marks H3K4me1 and H3K36me3. **(C)** Suppressed genes are DNA-hypermethylated at least in part thanks to the enzymatic activity of histone methyltransferases (KTMs) and histone deacetylases (HDACs). These genes express the repressive marks H3K9me3 and H3K27me3. **(D)** Cancer-associated DNA hypomethylation that affects the promoter of tumor suppressor genes originates from the enzymatic activity of KDMs and HATs activated by mutation. **(E)** Aberrant DNA hypermethylation, which activates oncogene expression in hematologic malignancies, is due to activating mutations of KTMs, HDACs, and DNMT3A that is normally active in embryonic/progenitor cells only. DKK1, Dickkopf-related protein 1; H3/H4, histone 3 or 4; sFRP, Secreted frizzled-related protein 1.

DNA methylation is essential for normal development, where it is associated with other pivotal epigenetic processes such as X-chromosome inactivation, genomic imprinting, and suppression of repetitive elements. This methylation mainly includes *de novo* methylation that sets up DNA methylation patterns in early development [mediated by DNA methyltransferase (DNMT) 3A/B], and maintenance methylation that copies methylation patterns to the daughter strands during DNA replication (mediated by DNMT1) (Trowbridge et al., 2012). In the present manuscript, only DNA methylation occurring at gene promoter, i.e., at the nucleotide 5-methylcytosine of promoter region, will be considered. Such DNA methylation alters the ability of a gene to interact with transcription factors through DNA conformational changes. Normal inactive genes are silenced by this mechanism (Figure 1A). Instead, gene suppression occurs through DNA hypermethylation (Figure 1B), which can be induced or reinforced by histone methylation, histone deacetylation, and histone dephosphorylation mediated, respectively, by histone lysine methyltransferases (KTMs), histone deacetylases (HDACs), and histone serine/threonine/tyrosine phosphatases (Yang et al., 2019). Conversely, transcriptionally active genes are demethylated (Figure 1C). DNA demethylation can be mediated by DNA demethylases and DNA glycosylases (Wallace and Obeng, 2023), or induced by histone demethylation, histone acetylation, and phosphorylation mediated respectively, by histone lysine demethylases (KDMs) histone acetyltransferases (HATs), and histone serine/threonine/tyrosine kinases. Upregulation or downregulation of small non-coding RNA molecules like microRNAs (miRNAs) can modify gene response to DNA methylation, post-transcriptionally; miRNAs modulate gene expression by targeting protein-coding RNAs (Ismail et al., 2023).

### 2.2 Changes in histone conformation: regulation of chromatin accessibility

Five families of histones are found in eukaryotic cells, namely, H1/H5, H2A, H2B, H3, and H4. H1 and H5 histones (linker histones) lock the DNA into place by binding the DNA entry and exit sites and the nucleosome, whereas the other histone families (core histones) form the nucleosome core. Chromatin accessibility (chromatin status), determined by histone conformation, affects gene transcriptional activity. The more thickening of the chromatin, the more suppressed are the genes (Audia and Campbell, 2016).

Several types of histone modifications have been discovered. Acetylation, methylation, phosphorylation, and ubiquitylation are the well-understood, while GlcNAcylation, citrullination, crotonylation, sumoylation, isomerization, ADP-ribosylation, deamination, formylation, propionylation and butyrylationare more recent discoveries that have yet to be thoroughly investigated. Thus, several families of enzymes are involved in chromatin status regulation.

Phosphorylation occurs on all core histones, with differential effects on each. Phosphorylation of histone H3 and histone H2A are involved in chromatin compaction and the regulation of chromatin structure and function during mitosis. Phosphorylation of H2AXand H2B serves as a recruiting point for DNA damage repair proteinsfor the former and is found to facilitate apoptosis-related chromatin condensation, DNA fragmentation, and cell death for the latter (Zhang and Zhang, 2020).

Histone ubiquitination regulates gene transcription and DNA repair. Despite the fact that all histone core proteins can be ubiquitylated, H2A and H2B are most commonly and are two of the most highly ubiquitylated proteins in the nucleus. Monoubiquitylated H2A is associated with gene silencing, whereas H2B is associated with transcription activation. Monoubiquitinand K63-linked polyubiquitin chains typically modulate protein function, localization, or interaction with DNA and/or other proteins. Protein ubiquitination levels are dynamically regulated by the opposing activity of deubiquitinating enzymes (DUBs) (Jeusset and McManus, 2019).

KTMs of the polycomb (PcG), the trithorax (trxG) groupsare the major regulators of histone methylation status. They bind to specific regions of DNA resulting in gene silencing (PcG) or activation (trxG). PcG form DNA-binding protein complexes named polycomb repressive complexes (PRCs), which mainly include: *i*) PRC1, consisting of the core subunits Ring1B, PH1, CBX4, and BMI1 (Smith et al., 2011); and *ii*) PRC2, whose composition is variable and which is most often made of the core subunits SUZ12, RBBP7, Jarid2, and EED, and EZH2, a major KTM for H3K27. Histone methylation and demethylation involves modification of certain amino acids in a histone protein by strategic addition or removal of one, two, or three methyl groups thus turning the genes in DNA “off” and “on”, respectively. For example, monomethylation of lysine 4 of histone 3 (H3K4) is associated with gene activation; activepromoters are marked by dimethylated (H3K4me2) and trimethylated forms (H3K4me3) of H3K4, whereas H3K9me3 and H3K27me3 are repressive marks. KTMs can form complexes with KDMs for the removal of othermethyl-marks. Experimental evidence indicates that deregulations of KTM/KDM activities are part of carcinogenic mechanisms in lung, kidney, prostate, breast, and blood cancers (Hung et al., 2010).

Similarly, Histone acetylation is involved in cell cycle regulation, cell proliferation, and apoptosis and may play a vital role in regulating many other cellular processes, including cellular differentiation, DNA replication and repair, nuclear import and neuronal repression. HATs and HDACs respectively add and remove acetyl groups from lysine residues on the histone N-terminus. Histone acetylation and deacetylation allow interconversion between repressive and permissive chromatin domains in terms of transcriptional competence, respectively. This is attained, by suppressing and creating docking sites for the binding of regulatory proteins (Hou et al., 2012).

## 3 Epigenetic changes in cancer: silencing of tumor suppressor genes and activation of oncogenes

Recent studies have provided new insights in the mechanistic involvement of epigenetic changes in carcinogenesis.

### 3.1 Alteration of DNA methyaltion and cancer

Aberrant DNA methylation has been accepted as a feature across multiple cancer types. Changes affecting DNA methylome in cancer include a loss in DNA methylation, hypomethylationand hypermethylation (Hung et al., 2010) affecting specific groups of genes (Figures 1D, E). For example, BRCA1 and RAD51C methylation is found in ovarian and breast cancers, and APC methylation in gastrointestinal cancers. Hypermethylation of CDKN2A/p16 promoter leads to inactivation of this gene in oesophageal adenocarcinoma (Geissler et al., 2024). Certain proto-oncogenes can also be activated by loss of promoter methylation, as, BCL2 in B-cell CLLand NFATC1 in B-cell CLL (Wolf et al., 2018). Silencing of the *TERT* oncogene is, in contrast, associated with promoter region hypomethylation.

Interestingly, about 75% of key developmental regulators, i.e., oncogenic pathway regulatory genes, that are hypermethylated in the context of bivalent chromatin in both embryonic and adult stem/progenitor cells, and which allow these cells to maintain their stemness, would also be silenced in various cancer cell types. Thus, preferential DNA hypermethylation targeting these subset of oncogenic pathway regulatory genes may contribute to the stem-like state of cancer cells like leukemia stem cells (LSCs) (Grzasko et al., 2012). DNA methylation of tumour suppressors can itself be mutagenetic and induce genetic alterations. Such mutations may explain at least in part regulatory gene silencing in cancer cells.

### 3.2 Aberrant histone modifications and cancer

Aberrant histone ubiquitination can drive oncogenesis by altering the expression of tumor suppressors and oncogenes, misregulating cellular differentiation and promoting cancer cell proliferation (Jeusset and McManus, 2019). These aberrations exist in many cancer types. Notably, global decreases in H2AK119ub1 levels occur in prostate cancer, while decreased H2BK120ub1 levels are frequently observed in breast, lung, and colorectal cancers relative to normal (Jeusset and McManus, 2019). Moreover, the genes encoding histone E3 ubiquitin ligases and DUBs are also frequently altered in cancers, and many of the enzymes possess tumor suppressor (e.g., BAP1 and RNF20) or oncogenic potential (e.g., BMI1 and USP22) (Melo-Cardenas et al., 2018), identifying possible mechanism(s) accounting for the aberrant histone ubiquitination levels observed within those cancers.

An imbalance in the equilibrium of histone acetylation is associated with tumorigenesis and cancer progression. Indeed, dysfunctions of Histone deacetylase (HDAC) enzymes and altered level of acetylation are linked to various cancers including cholangiocarcinoma (Peng and Seto, 2011). HDACs play a vital role in the epigenetic regulation of gene transcription and expression through their effects on the chromatin compaction state and affecting the stability of other cellular target proteins. The overexpression of HDACs have been reported in various solid and hematological cancers (Shankar and Srivastava, 2008) affecting diverse cellular mechanisms such as proliferation, cell death, metastasis, autophagy, metabolism, and ciliary expression.

Many of the methyltransferases and demethylases responsible of the methylation or demethylation of histone are amplified, overexpressed, deleted, misregulated, rearranged, or mutated on cancer. The partial duplication of MLL1’s set KMT domain corroborates a role for MLL1 in cancer, showing in this case, increased HoxA gene expression associated with increased H3K4me3 marks at the promoter (Dorrance et al., 2006). In the case of MLL2/3 loss-of-function mutations, cancers may alter H3K4 methylation states. The first reported lysine demethylase (KDM) enzyme specific to H3K4 and H3K9 residues, LSD1 (KDM1A), is a classic oncogene (Lau et al., 2011) and is overexpressed in many types of cancer. About KDM5B, ithas a putative tumor-suppressive functionin metastatic melanoma. With respect to the G9a KMT, both deletion or lowered expression and increased expression have been observed in cancer. For H3K9-specific demethylases, KDM3A and KDM4 are often amplified or highly expressed, whereas attenuated expression of KDM4A has been observed in cancer (Lau et al., 2011).

Histone phosphorylation is essential for maintaining the equilibrium of kinase-phosphatase at kinetochore to refrain from chromosomal instability and cancer.

Phosphorylation occurs on hydroxylated aminoacids tyrosine, serine, and threonine.H2Bser32p is extensively phosphorylated by RSK2 kinase in skin cancer cells (Lau et al., 2011).In addition, androgen stimulation protein kinase C-beta (PKCβ) and protein kinase C related kinase 1 (PRK1) phosphorylate H3tre6 and 11 respectively in prostate cancer cells (Metzger et al., 2010). Likewise, loss of heterochromatin protein 1α (HP1α) from chromatin leads to constitutive activation of the Janus kinase 2 (JAK2 which phosphorylates H3tyr41) signaling pathway including oncogene imo2, leading to oncogenesis (Shanmugam et al., 2017). Equally, downregulation of dual specificity phosphatase 22 (DUSP22) expression, elevation of phosphorylation of the H2AX histone (p-H2AX), reduced level of Histone H3 at Ser10 (H3S10), and the phosphorylation of T-LAK cell-originated protein kinase (TOPK) at Y74 and Y272 are observed in colorectal cancer (Qin et al., 2020).

## 4 Epigenetic regulation, in normal hematopoiesis and HMs

### 4.1 Normal hematopoiesis

Recent experimental data suggest that epigenetic regulators play important roles in normal hematopoiesis. For instance, the PRC1 member BMI1 which promotes hematopoietic stem cell (HSC) development from embryonic stem cells, is required for efficient HSC maintenance, and confers HSCs with resistance to oxidative stress through negative regulation of ROS signaling (Nakamura et al., 2012). Similarly, the PRC2 member EZH2 activity is required to prevent HSC senescence. The HAT activity of the multidomain protein MOZ (monocytic leukemia zinc finger) was reported as critical for HSC development, maintenance, proliferation, and differentiation (Yang, 2015). Furthermore, the recruitment of the HAT CREB binding protein (CBP) is pivotal for the function of the hematopoietic transcription factor GATA-1. More recent studies indicate that ZBP-89, a novel GATA-1, physically associates with the HAT p300 to play key roles in erythroid differentiation (Woo et al., 2011). Interestingly, both PRC1 and 2 can recruit DNMT1, and PRC2 can recruit HDAC1 and 2, indicating that epigenetic processes collaborate for HSC maintenance, and probably for maintenance of LSCs originating from them as well.

Conversely, the importance and the roles of some epigenetic regulators (mainly HATs) in normal hematopoiesis are controversial. Data discordance originated mainly from loss-of-function studies, such as the report from Kimbrel and collaborators, which indicates that inactivating mutations affecting HAT activity have only minimal effects on hematopoiesis; in addition and quite surprisingly, the nullizygosity of CBP and p300 resulted in significant defects in hematopoiesis even in mutants with active HAT enzymatic activity, suggesting that these enzymes are dispensable for this process (Kimbrel et al., 2009). Thus, we understand the importance of studying the role played by epigenetics in the biology of hematological malignancies.

### 4.2 Epigenetic alterations and hematologic malignancies

Epigenetic regulation controls the expression of genes that affect HSC survival, proliferation, and stemness, andconsequently, leukemogenesis. Alterations of DNA methylation and other epigenetic processes due to leukemogenic mutations result in a block of differentiation; these alterations enhanced self-renewal in transforming cells, even in absence of microenvironment. Epigenetic alterations resulting in DNA methylome changes in cancer are complex. Alterations of epigenetic regulation reported in various HMs are summarized in Table 1.

**TABLE 1:** Alterations of epigenetic regulation reported in various HMs.

	Alterations observed	Hematopoietic malignancies
Regulators methylating DNA		
DNMTs	DNMT1 overexpression	Leukemias (Shanmugam et al., 2017)
	DNMT3A mutations	MDS, AML (Zhang et al., 2021; Ismail et al., 2023), lymphomas
	Gene hypermethylation	ALL [Gruszka et al., 2019], AML [Sallman et al., 2021], CLL, MCL, DLL, BL (Urbano et al., 2019)
HDACs	Overexpression	CLL (Xie and Chng, 2014; Liu et al., 2020)
KTMs	Silencing/deletion	AML, CML, FL, DLL
	Overexpression	AML (Shanmugam et al., 2017), CML, ALL (Billot et al., 2011), APL (Nishioka et al., 2010), MLL, MM (Audia and Campbell, 2016; Kalinkova et al., 2023), HL (Zhang and Zhang, 2020)
miRNAs	Downregulation	AML (Lau et al., 2011), NFL (Yang, 2015), BL (Woo et al., 2011)
Regulators demethylating DNA		
HATs	Fusion proteins with HAT activity	Leukemias (Weng et al., 2018)
	Inactivating mutations	B-cell lymphoma
KDMs	Overexpression	Leukemias

ALL, acute lymphocytic leukemia; AML, acute myeloid leukemia; APL, acute promyelocytic leukemia; BL, Burkitt lymphoma; CLL, chronic lymphocytic leukemia; CML, chronic myeloid leukemia; DLCL, diffuse large B-cell lymphoma; DNMTs, DNA, methyltransferases; FL, follicular lymphoma; HATs, histone acetyltransferases; HDACs, histone deacetylases; HL, Hodgkin’s lymphoma; KDMs, histone demethylases; KTMs, histone methyltransferases; MCL, mantle-cell lymphoma; MDS, myelodysplastic syndromes; miRNAs, microRNAs; MLL, mixed lineage leukemia; MM, multiple myeloma; NFL, t(14;18)-negative follicular lymphoma; PcG, polycomb group; PCGF6, polycomb group ring finger 6; trxG, trithorax group.

#### 4.2.1 DNA hypomethylation and anticancer drugs targeting methylation

Despite endeavours of several researchers, little is known about DNA demethylases and DNA glycosylases, major enzymes involved in DNA demethylation, in HMs. Recent reports have indicated that the pivotal component of the DNA base excision repair pathway 8-oxoguanine glycosylase (OGG1), which removes 8-oxoguanine nucleotides from DNA (thereby suppressing mutagenesis and rescuing cells from apoptosis) was significantly downregulated by the RUNX1-ETO leukemia fusion protein in both normal hematopoietic progenitor cells and in cells from AML patients (Liddiard et al., 2010). A study addressing OGG1 polymorphism reported that the presence of some OGG1 alleles (R229Q mainly) may be predictive of an increased cell susceptibility to enter in malignant transformation (He et al., 2022); polymorphism of the enzyme has been also associated with increased risk for developing AML in children (Stanczyk et al., 2011).

Some studies have indicated that other mechanisms inducing DNA demethylation, such as histone demethylation mediated by KDMs and histone acetylation mediated by HATs, play key roles in LSC development and maintenance. Interestingly, a recent study suggested that the H3K9 demethylase KDM3B represses leukemia cell differentiation, and is upregulated in blood cells of acute lymphoblastic leukemia (ALL) patients (Kim J. Y. et al., 2012). KDM3B displayed histone H3K9-me1 and 2 demethylase activity and induced the expression of the leukemogenic oncogene lmo2 through interdependent actions with HATs. In addition, KDM3B is downregulated during all-trans-retinoic acid (ATRA)-induced differentiation of HL-60 APL cells (Kim J. Y. et al., 2012). Inactivating mutations of PRC2 components EZH2, SUZ12, EED, and Jarid2 have been recently reported in various myeloid neoplasms. Furthermore, The HAT activity of MOZ generates fusion genes with other HATs, such as CBP and p300 in AML; the activities of the resulting fusion proteins lead to repressed differentiation, hyper-proliferation, and self-renewal of transforming myeloid progenitors. Moreover, DNA hypomethylation is associated with the overexpression of the oncogenic transcription factor Myc in lymphoma (Wolyniec et al., 2012). Taken together, these findings indicate that DNA hypomethylation plays a pivotal role in the pathogenesis of HMs. It is thus envisaged that further studies may reveal novel pharmacological targets for treatment.

Epigenetic regulators of chromatin status appear as striking therapeutic targets because of the potentially reversible characteristics of histone modifications (Chandra, 2021).Of particular interest as far as therapy is concerned, many reports indicate that DNA methylating agents targeting KTMs or HATs are promising as future anticancer drugs. DNA methylating agents aim at suppressing the expression of oncogenic genes aberrantly activated by DNA hypomethylation in cancer cells. Efforts towards the development of specific KDM inhibitors (KDMi) are in progress. In particular, targeting of the Jmj domain, a cofactor essential for the demethylase activity of KDMs, has yielded interesting results. For instance, Zn-ion ejecting compounds like disufiram and ebselen were reported to inhibit JmjD2A by disruption of its Zn-binding site (Hua et al., 2010). Interestingly, LSD1 (lysine-specific histone demethylase 1) shows homology in its catalytic site with the flavin-dependent amine oxidases (MAO) that are targets for the treatment of neurological disorders. MAO inhibitors, such as tranylcypromine and phenelzine, inhibit LSD1 activity (Karakurt et al., 2012). In AML, LSD1 inhibition reactivates the ATRA differentiation pathway. Similarly, HAT inhibitors (HATi) display promising therapeutic properties in various cancers. For example, garcinol, a potent HATi, radiosensitizes A549 lung and HeLa cervical carcinoma cells by inhibiting non-Homologous end joining, and the green tea catechin epigallocatechin-3-gallate inhibits EBV-induced B-lymphocyte transformation via suppression of p65 acetylation (Hung et al., 2010).

#### 4.2.2 DNA hypermethylation and anticancer drugs targeting methylation

Experimental data indicate that aberrant DNMT activity and DNA hypermethylation observed in LSCs are involved in leukemogenesis. For instance, conditional knockout of DNMT1 blocked the development of leukemia and its haploinsufficiency delayed leukemogenic progression and impaired LSC self-renewal without altering normal hematopoiesis (Trowbridge et al., 2012), suggesting that LSCs depend on DNA methylation-mediated silencing of bivalent domains to enforce gene suppression.

Most KTMs involved in hematopoiesis have been reported to play key roles in leukemogenesis. For instance, Suv39 h1 and Suv39 h2 mediate a direct trimethylation of H3K9 at pericentric heterochromatin that causes bone marrow cell immortalization (Goyama et al., 2010). Furthermore, the AML fusion protein NUP98-NSD1is an active H3K36 KTM (Liu et al., 2020), and the multiple myeloma (MM) SET domain (NSD2 or MMSET) is a KTM involved in the t (4; 14)(p16; q32) translocation, a recurrent chromosomal translocation present in MM andassociated with poor prognosis. MMSET switches global histone methylation and recruits HDAC1 and 2 in t (4; 14) MM cells (Xie and Chng, 2014), resulting in sustained DNA hypermethylation. Overexpressions of HDAC1, 2, 3, 6, 7 and 9 have been reported in many leukemias, including CLL, where it contributes to disease pathogenesis (Wang et al., 2011).

Aberrant DNA hypermethylation may contribute to leukemogenesis by silencing tumor suppressor genes. For instance, leukemogenic inactivating mutations of the IDH2 gene have been associated to both DNA and histone hypermethylation in AML (Figueroa et al., 2010). DNA hypermethylation may be sustained and coordinated by aberrantly activated PcGs in cancer cells; the latter KTMs cause histone methylation directly, as well as DNA hypermethylation and histone deacetylation indirectly through the recruitment of DNMT1 (Bhatla et al., 2012) and HDAC1/2. As also observed in stem/progenitor cells, many tumor suppressor genes targeted by PcG, such as WT1, RARβ, KLF4, ID4, GATA3, CHD5, and SPI1, accumulate DNA methylation at their promoters in cancer (Issah et al., 2021). This phenomenon occurs in several HMs, including ALL, CML, diffuse large B-cell lymphoma, follicular lymphoma, and Burkitt lymphoma, suggesting that these mutations are a common phenomenon in cancers. Pharmacological targeting of these changes may yield good results for therapy.

DNMT inhibitors (DNMTi), such as decitabine (5-aza-2′-deoxycytidine) and 5-azacytidine have been recently approved for the treatment of MDS, CML, and AML, whereas other DNMTi have entered clinical trials. Overall, DNA hypomethylating agents developed for cancer aim at restoring the expression of tumor suppressor genes silenced by aberrant DNA hypermethylation. They target DNMTs, KTMs, or HDACs. For instance, decitabine, which is currently used for high-risk MDS patients not eligible for HSC transplantation, significantly reduces global methylation in AML cells, and improves response rates as compared to standard therapies in AML patients (Kantarjian et al., 2012). This drug induces apoptosis in AML cells through intracellular reactive oxygen species generation; however, decitabine has important adverse effects, including bone marrow toxicity (Greenberg et al., 2012). This pitfall has been partially fixed by using low doses of the drug in combination with classical anticancer drugs, which represents a better therapeutic approach than monochemotherapy, particularly against chemoresistant cancers (Greenberg et al., 2012). In addition, the KTM inhibitor (KTMi) chaetocin, which inhibits Suv39 h1 and G9a, successfully blocked the hypermethylation of tumor suppressor genes in AML cells, and displayed antimyeloma activity by triggering oxidative stress in MM cells (Han et al., 2017). Furthermore, several HDACi have been developed, some of which are already in use in cancer therapy. Interestingly, novel HDACi showing potent antileukemic activity in myeloid cell lines and primary AML blasts at low micromolar concentrations have been developed (Hackanson et al., 2012).

Moreover, experimental evidence indicates that besides activating mutations, PcG overexpression resulting in DNA hypermethylation is also due to PcG regulator silencing or loss. PcG regulators silenced in cancer include miRNAs, whose downregulation is a hallmark of various cancers, including t (14; 18)-negative follicular lymphoma and Burkitt lymphoma 48]. In addition, the miR-137 and miR-214, among others, modulate cancer cell differentiation through the control of EZH2 protein levels, and the downregulation of miR-200 c, miR-203, and miR-183 ensures the expression of BMI1 in mouse embryonic stem cells, and in various cancer cell lines (Anderson and Guttilla Reed, 2020), contributing to the maintenance of the stemness of these cells. Non-coding RNA -based therapies preventing the translation of mutation-activated DNMTs are in progress. Potential miRNA-based drugs include miR-29b, whose enforced expression in AML cells results in a significant reduction in the expression of DNMT1 and DNMT3A/B at both RNA and protein levels (Yang et al., 2019). Synthetic miR-29b oligonucleotides may represent effective hypomethylating compounds in AML.

## 5 Epigenetic regulation, signaling pathways, and HM risk factors

### 5.1 Risk factors associated with HMs

HMs are multifactorial diseases and in many cases the etiology is not well understood. Population-based studies, as well as recent clinical and experimental reports suggest that in obese individuals and diabetic patients, particularly but not exclusively, chronic metabolic syndrome and associated disorders, such as insulin resistance, dyslipidemia, chronic inflammation, and oxidative stress, are risk factors for developing HMs, including CML and ALL (Ma et al., 2020), MM (Corre et al., 2021), non-Hodgkin’s and Hodgkin’s lymphomas (Wallin and Larsson, 2011), but also various solid cancers (Wu et al., 2018). Major mediators of metabolic syndrome include: *i*) the receptor of the adipokine leptin and type 1 and 2 receptors of pro-inflammatory cytokines which mediate their effects mainly through the oncogenic Janus kinase (JAK)/signal transducer and activator of transcription (STAT) pathway, and JAK-mediated activation of the mitogenic Ras GTPases/mitogen-activated protein kinases (MAPKs)/ERKs (extracellular signal-related kinases) pathway, and survival pathways activated by the phosphatidylinositol 3-kinase (PI3K)/Akt such as mammalian target of rapamycin (mTOR) signaling pathway (Oliveira et al., 2012); *ii*)growth signals mediated by tyrosine kinase receptors like insulin receptor isoforms A (IR-A) (Aldhafiri et al., 2012), insulin-like growth factor receptor type 1 (IGF-1R) (Karakurt et al., 2012), and epidermal growth factor receptor (EGF-R) (Weng et al., 2018), which induce insulin receptor substrate-mediated triggering of the Ras/MAPKs/ERK1/2 pathway, and survival pathways activated by PI3K/Akt such as mTOR (Gallagher et al., 2012), β-catenin (triggered by inactivation of glycogen synthase kinase 3β or GSK3β protein) (A Portilho et al., 2021), and pathways activated by suppression of forkhead box O (FoxO) transcription factors (Tzivion et al., 2011); *iii*) and pro-inflammatory signaling pathways like, nuclear factor κB (NF-κB), CCAAT-enhancer-binding proteins (c/EBPs), and activating transcription factor (ATF)/cAMP response element-binding protein (CREB) family of molecules (Wang et al., 2018), among others. NF-κB produces both pro- and counter-inflammatory factors, including cytokines like interleukin 1β (IL-1 β), IL-6, IL-12, and tumor necrosis factor α (TNF-α), chemokines like CCL2 and CCL5, adhesion molecules like intercellular adhesion molecule 1 (ICAM1) and P-selectins, inhibitors of NF-κB signaling like IκB-α and A20, the inhibitor of macrophage activation CD200, and the inhibitor of virus proliferation interferon (IFN)-β (Newton and Dixit, 2012).

Interestingly, epigenetic regulators such as HDAC5 and histone 2A.v (H2AV) are also produced during mononuclear cell activation, concomitantly with well-known pro-inflammatory signaling transcriptional factors such as CCAAT-enhancer-binding proteins (c/EBPs), and activating transcription factor (ATF)/cAMP response element-binding protein (CREB) family of molecules (Wang et al., 2018).

### 5.2 Epigenetic mechanisms in signaling pathways associated with HM risk factors

#### 5.2.1 Pro-inflammatory signaling pathways in HMs: the case of NF-κB

Constitutive activation of NF-κB has been reported in several HMs. A majority of primary myeloma patient samples and cell lines display an elevated expression of NF-κB target genes, including TRAF3, CYLD, BIRC2/BIRC3, CD40, NFKB1, or NFKB2, suggesting that addiction to NF-κB signaling is frequent in myeloma (Vrábel et al., 2019). In addition, NF-κB gene expression is often associated with genetic or epigenetic alterations, indicating a possible role of these processes in the pathogenic effects of the pro-inflammatory signaling pathway in myeloma. In another study, non-coding RNA expression profiling in bone marrow derived CD138 (+) MM cells have revealed a MM-specific miRNA signature characterized by a significant downregulation of miRNA-15a/-16 and upregulation of miRNA-222/-221/-382/-181a/-181b (Zhang et al., 2018). Functional studies revealed that miRNA-15a and -16 post-transcriptionally regulate the proliferation and growth of MM cells *in vitro* and *in vivo* by inhibiting Akt, MAPKs, and the NF-κB-activator MAP3KIP3. A study addressing the role of the pro-inflammatory transcription factor CREB in AML cell abnormalities (Takao et al., 2021) revealed an overexpression of its target genes, such as NF-κB, JAK1, STAT3, the cell cycle regulators cyclin A1, B1, D, and the apoptosis regulator Bcl-2; survival signaling pathways, including Akt, mTOR, and ERK, were also concomitantly activated. Interestingly, CREB activation resulted from silencing by hypermethylation of its regulatory genes miR-34b and miR-34 c genes.

Furthermore, PcG-mediated miRNA silencing and NF-κB activation were reported among the pathogenic events mediating adult T cell leukemias caused by human T cell leukemia virus type 1 (HTLV-1) (Yamagishi et al., 2012), providing evidence for a direct epigenetic control of inflammatory responses involved in leukemia pathogenesis. Except for CLL, most B-cell malignancies show frequent inactivation of the NF-κB inhibitor A20 (Frenzel et al., 2011). In addition, Aiolos, a member of Ikaros family of transcription factors activated by NF-κB, is upregulated in B-CLL cells by chromatin status modulation-induced DNA demethylation (Billot et al., 2011). Similarly, epigenetic silencing of the tumor suppressor death-associated protein kinase 1 (DAPK19, which controls NF-κB- responsive genes, was recently reported in heterogeneous primary AMLs, HMs associated with a poor prognosis (Shanmugam et al., 2012). Altogether, these findings emphasize the pivotal role of NF-κB in leukemogenesis, and indicate that this pro-inflammatory factor is activated by epigenetic regulation in HMs originating from primary lymphoid organs such as leukemias and myeloma. Inhibition of JmjD3, a member of the JmjC family of KDMs, silenced various NF-κB key genes in monocytic cell lines (Das et al., 2012); the DNMTi decitabine and the HDACi trichostatin and valproic acid rapidly inhibited NF-κB aberrant expression in malignant myelobasts *in vitro* and *in vivo* (Quagliano et al., 2020). Reports published in 2012 have suggested an involvement of NF-κB in the pathogenesis of HMs originating from secondary hematopoietic organs. For instance, epigenetic activation of NF-κB-related genes, including CARD11, CD79B and MYD88, have been reported in diffuse large B-cell lymphomas such as mucosa-associated lymphoid tissue (MALT) lymphoma (Liu et al., 2012). Epstein-Barr virus (EBV) associated HMs, the PRC1 member BMI1, which is a repressor of the tumor suppressor complex p16INK4a/p14ARF (Martinelli et al., 2011), and several other epigenetic regulators modulate cellular gene expression programs by affecting cellular levels of miR-146a and miR-155 via the NF-κB pathway (Markopoulos et al., 2018). Furthermore, miR-125a and miR-125b constitutively activate the NF-κB pathway by targeting A20 in diffuse large B-cell lymphoma (Kim S. W. et al., 2012).

#### 5.2.2 Metabolic syndrome, signaling pathways and HM risk factors: Ras/MAPK and PI3K/Akt

Emerging evidence indicates that Ras/MAPK signaling pathway can mediate leukemogenic effects through epigenetic mechanisms. For instance, Hesson and collaborators have reported that RASSF6 and RASSF10, tumor suppressor genes of the Ras-association family (RASSF), are hypermethylated in leukemia cells (Liu et al., 2020). Interestingly, such epigenetic silencing of RASSF6 and RASSF10 appeared to be extremely frequent in childhood leukemia, and were reversed by treatment with the hypomethylating drug decitabine, underlying the potential of epigenetic drugs in the treatment of HMs. Similarly, p53 has recently been reported to methylate RASSF1A gene in a murine model of ALL (Zhang et al., 2012). A study addressing the role of the gene associated with increased risk for AML Gfi1 (growth factor independence 1) in mouse have revealed that K-Ras driven myeloproliferative disorders induce mutations in Gfi1 gene, whose associated loss of functions induces histone modifications resulting in the expression of Hoxa9 gene and other genes involved in the development of leukemia (Khandanpour et al., 2012). Silencing of RASSFs through epigenetic regulation has also been reported in murine models and cancer cells from patient diagnosed with lymphomas and various solid cancers, including gliomas, adenomas, and carcinomas (Sun S. et al., 2022). Altogether, these findings indicate that Ras/MAPK can silence tumor suppressor genes and activate oncogene expression through epigenetic mechanisms.

Similarly, recent experimental data also indicate that PI3K/Akt signaling pathway can mediate carcinogenic transformation through epigenetic mechanisms in HMs. For instance, together with cellular senescence associated telomerase gene silencing, PI3K/Akt signaling is suppressed during the anticancer treatment-induced differentiation of human AML cell line 60 (HL60) through mechanisms involving H4 acetylation (Zhang et al., 2021). In a study addressing the role of IGF-1 in the pathogenesis of MM, the growth factor induced antiapoptotic effects on these malignant cells by suppressing the expression of the pro-apoptotic BH3-only protein Bim via epigenetic mechanisms (De et al., 2010). More specifically, IGF-1 decreased cellular levels of Bim gene product by increasing its proteasome-mediated degradation through a MAPK signaling-dependent mechanism, and the activation of the Akt pathway, as well as the resulting inactivation of the transcription factor FoxO3a, reduced the expression of Bim gene through promoter region unmethylation (which causes gene inactivation), H3K9 dimethylation and acetylation, and through post-transcriptional RNA interference. Treatment with either or both the hypomethylating drugs decitabine (DNMT inhibitor) and LBH589 (HDAC inhibitor) induced a significant upregulation in the expression of Bim.

Kikuchi and collaborators (Kikuchi et al., 2011) have demonstrated that the HAT GCN5 regulates the activation of PI3K/Akt pathway in B cells exposed to oxidative stress induced by exogenous hydrogen peroxide (H2O2), by controlling the expression of genes involved in the activation of the pathway, such as spleen tyrosine kinase (Syk) and Bruton’s tyrosine kinase (Btk). It would be interesting to determine whether such mechanism also apply in transforming cells during the pathogenesis of HMs. In addition, the hypermethylation of genes of Eph family, receptors of the tumor suppressor EPHB4 involved in normal hematopoietic development, may contribute to ALL pathogenesis, as revealed by a study in both leukemia cell lines and primary ALL bone marrow samples (Kuang et al., 2010). Moreover, chemoresistance of CML and chronic eosinophilic leukemia (CEL) to anticancer treatment with the multi-targeted tyrosine kinase inhibitor imatinib may occur via hypermethylation of the “phosphatase and tensin homolog deleted on chromosome 10” (PTEN) gene involved in the dephosphorylation (silencing) of AKT, ERK and STAT5 signaling pathways (Nishioka et al., 2010). Methylating epigenetic drugs restored PTEN expression. In a study in murine bone marrow where comparable observations were reported, PTEN suppression activated PI3K/AKT/mTOR signaling through interactions with KTMs of the PcG group (Yoshimi et al., 2011).

### 5.3 Epigenetic changes in HMs trigger aberrant oncogenic signaling pathway expression

**FIGURE 2:**
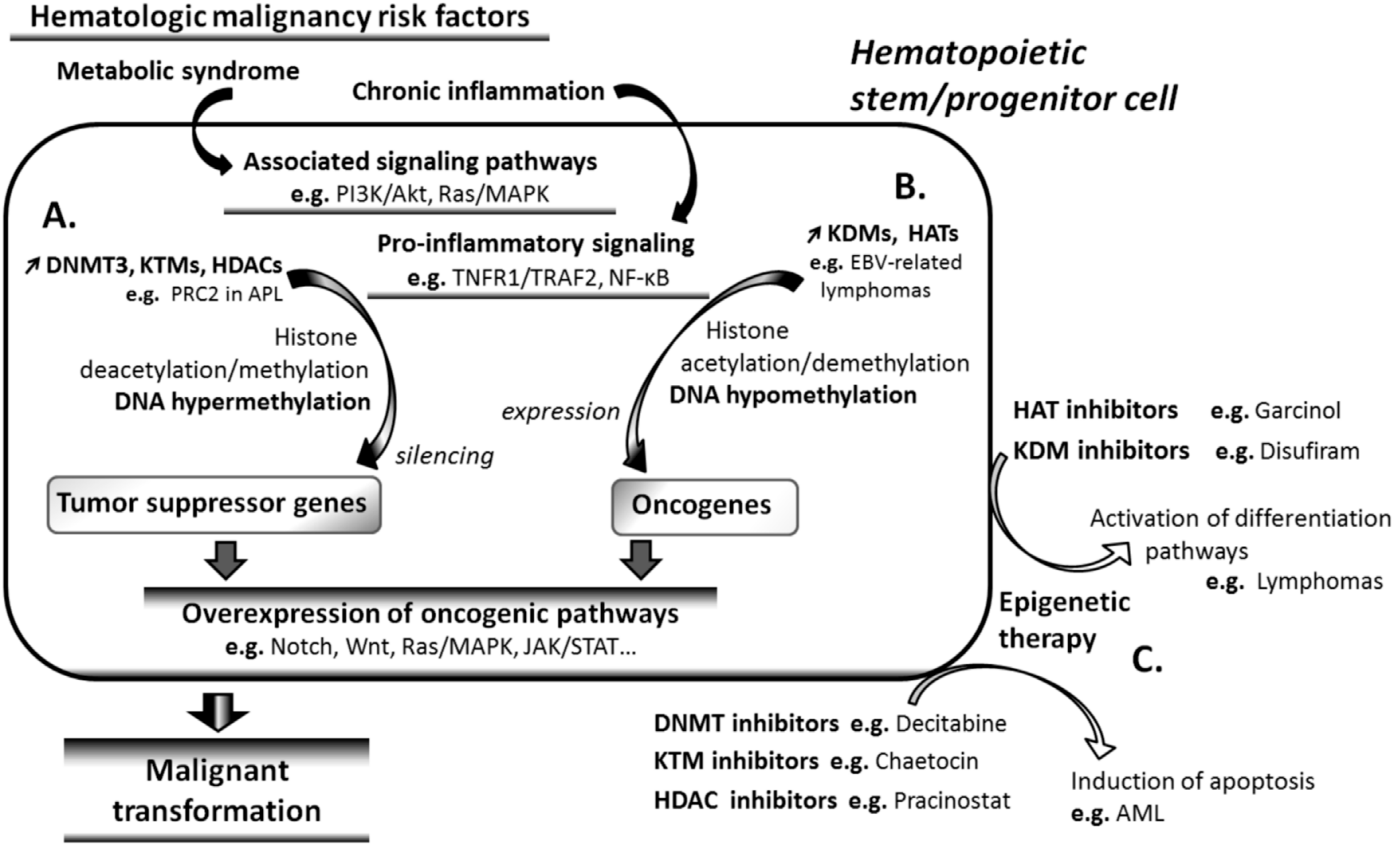
Cancer risk factors and carcinogenesis. Signaling pathways associated with risk factors of hematologic malignancies (HMs) such as metabolic syndrome and chronic inflammation cause tumor suppressor genes repression by inducing DNA hypermethylation **(A)**, as well as oncogene expression through DNA demethylation **(B)**. Both effects result in the overexpression of oncogenic signaling pathways and in the subsequent malignant transformation of the affected cells. **(C)** Epigenetic drugs reverse the effects of risk factors on gene methylation. DNA hypomethylating agents induce apoptosis, whereas DNA methylating agents reactivate differentiation pathways such as all-trans-retinoic acid pathway, in transforming cells. AML, acute myeloid leukemia; APL, acute promyelocytic leukemia; EBV, Epstein-Barr virus; JAK, Janus kinase; MAPK, mitogen-activated protein kinase; PI3K, phosphatidylinositol 3-kinase; PRC2, polycomb repressive complex 2; STAT, signal transducers and activators of transcription; TNFR1, TNF receptor 1; TRAF2, tumor necrosis factor receptor associated factor 2.

Clinical and experimental data indicate that several signaling pathways that are active during embryonic development, such as Wnt, Notch, JAK/STAT, Ras/MAPK, and Akt/mTOR pathways (Roy and Ghosh, 2021), are involved in adult HSC maintenance and LSCs development. Aberrant hypermethylation of the regulatory genes of these pathways has been reported (Kalinkova et al., 2023), and a growing body of evidence indicates that epigenetic factors play a key role in the pathogenic overexpression of these signaling pathways in HMs (Figure 2).

#### 5.3.1 Anti-apoptotic and survival pathways: wnt and notch pathways

Genes encoding for Wnt antagonists are silenced by hypermethylation in several HMs, including genes such as secreted Frizzled-related proteins (sFRP), Dickkopf 3 homolog (DKK3), adenomatous polyposis coli complex (APC), human homolog of Dapper 1 (HDPR1), and Wnt inhibitory factor 1 (WIF1) in MM (Guo et al., 2017), MDS (Reins et al., 2010), AML (Gruszka et al., 2019), and CLL (Urbano et al., 2019). Such Wnt inhibitor silencing correlates with poor prognosisin MDS (Reins et al., 2010) and AML (Gruszka et al., 2019), and chemoresistance to classical anticancer drugs like imatinib mesylate in CML (Chen et al., 2022).

Evidence for direct silencing of Wnt inhibitors by epigenetic regulators has been provided by studies in lung cancer cells, where exposure to tobacco smoke condensate induced recruitment of PRC2 members EZH2, SUZ12, and PRC1 member BMI1, which remodeled histone marks at DKK1 gene promoter, including repressive mark H3K27me3 increase and active mark H4K16Ac decrease (Veneti et al., 2017). Not surprisingly, the specific Wnt inhibitors transcriptionally silenced by epigenetic regulation in HMs like CLL and ALL, among others, present a high interindividual variability (Hogan et al., 2011). Aberrant hypermethylation affected all regulatory HOX gene clusters and several Wnt inhibitors. These findings underline the importance of studying DNA methylome changes, and not individual genes, for pinpointing epigenetic predictors of classical anticancer drug chemoresistance and indicators of poor prognosis in patients.

Interestingly, findings from another recent study have strongly indicated that Wnt regulatory gene silencing may not be completely stochastic but result, instead, from reprogramming of epigenome orchestrated by the action of cancer causative agents through modifications of activation statuses of epigenetic “writers” and “readers”, mainly PRCs (Moskalev et al., 2012). Furthermore, several leukemia fusion proteins may induce the expression of Wnt molecules, such as E2A-PBX1 that induces the expression of Wnt16 in ALL (Verras et al., 2015). These findings emphasize the necessity for epigenetic therapy in cancer (see also section 5.2).

Aberrant activation of Notch signaling by epigenetic mechanism has also been widely reported. Besides DNA methylation at promoter region, a novel level of anti-apoptotic signaling control has been revealed by a study addressing mechanisms regulating Notch signaling pathway expression in T-ALL, a HM driven mainly by Notch signaling abnormal activation (Ma et al., 2012). Notably, switches in histone acetylation/deacetylation of pivotal Notch genes like Notch3 appeared as a key regulatory events, and therefore a potential therapeutic target. Aberrant transcription of Notch1 gene has been reported in T-ALL cells as well (Jeannet et al., 2010), and acetylation/deacetylation switch in Notch genes has also been reported in MM pathogenesis (Ismail et al., 2022). Experimental data indicate that a network of epigenetic and transcriptional regulators may control Notch signaling during both normal development and leukemogenesis (Jeannet et al., 2010; Yatim et al., 2012). It can be anticipated that such network is under the control of cancer causative agents, and therefore, that pharmacological targeting of ALL-associated epigenetic changes in Notch genes may represent a good therapeutic approach. Other studies have indicated that Notch signaling may mediate hypermethylation of the key erythroid factor globin transcription factor 1 (GATA1) gene which contributes to the ineffective erythropoiesis observed in late MDS (Hopfer et al., 2012); and epigenetic enhancement of Notch activation favored T cell lymphomagenesis in transgenic mice (Wang H. C. et al., 2012). Altogether, these observations suggest that epigenetic factors account, at least in part, for the aberrant expression of oncogenic signaling pathways in HMs.

#### 5.3.2 Growth and proliferation pathways: JAK/STAT and MAPK/ERK pathways

JAK proteins are intracellular non-receptor tyrosine kinases, which mediate pivotal and evolutionary conserved effects on cell growth and fate through downstream targets like STAT, Ras/MAPK, and PI3K/Akt signaling pathways (Zhong, 2016). Reports indicate that JAK2 is negatively regulated by the trxG H3K9 methyltransferase G9a in leukemia cells, and G9a knockdown directly increases JAK2-mediated phosphorylation of histone H3 on Tyr 41 (Y41) by inducing JAK2 expression, suggesting that G9a plays an upstream regulatory role in JAK2-H3Y41P-HP1α -mediated leukemogenesis (Son et al., 2012). Similarly, suppressor of cytokine signaling (SOCS) proteins have been reported to have negative regulatory roles on JAK2(V617F) transcription (Funakoshi-Tago et al., 2019). SOCS knockdown induced constitutive STAT5 phosphorylation in JAK2mu cells, and in myeloproliferative disorders SOCS2 hypermethylation was observed. Epigenetic silencing of SOCS1 and SOCS3 genes was observed in patients and murine models of chronic myeloproliferative disorders and AML expressing JAK2(V617F) mutation (Cooper et al., 2010), suggesting that JAK/STAT contributes to growth factor hypersensitivity induced by JAK2 mutations. Enforced expression of these genes in Lck-transformed murine leukemia cells (LSTRA cells) attenuated Lck-mediated malignant transformation through inhibition of STAT5 activity by inactivating phosphorylation (Cooper et al., 2010). In addition, the JAK2(V617F) mutation has been associated with Wnt regulatory gene epigenetic silencing in human myeloproliferative neoplasm pathogenesis (Bennemann et al., 2012); and the HDACi givinostat would induce its anticancer effects, at least in part, by selectively targeting cells bearing this mutation (Jenke et al., 2021), suggesting that genetic and epigenetic factors collaborate in the pathogenesis of these HMs. JAK inhibitors like ATP-competitive JAK (type I) inhibitors were developed to treat various HMs. Although they proved efficacy in both preclinical and clinical trials, dose-dependent toxicities were reported in patients (Lafave and Levine, 2012). Moreover, the trxG oncoprotein Ash2 L has been identified as a STAT signaling interactor in mixed-lineage leukemia (MLL) (Butler et al., 2011).

Triggering of several other growth, proliferation, and survival oncogenic pathways by epigenetic mechanisms has been reported in various HMs. Genomic screening for genes silenced by DNA methylation revealed that Ras-related protein RASD1 suppression correlates with dexamethasone resistance in MM (Rosenberg, 2022), aberrant methylation tends to affect more frequently genes involved in the MAPK signaling pathway in MDS and AML (Aldoss et al., 2019) as well, and Ras-association tumor suppressor genes RASSF6 and RASSF10 are frequently epigenetically inactivated in childhood leukemias (Liu et al., 2020). N-RAS or K-RAS genetic or epigenetic aberrant activations are associated with poor prognosis in plasma cell leukemia (PCL), an aggressive HM originating from secondary leukemic transformation of MM (Aldoss et al., 2019), and in ALL (Mullighan et al., 2011). Furthermore, NPM-ALK, the initiating oncogene of anaplastic large cell lymphomas, induces DNA damage and irreversibly arrests the cell cycle of hematopoietic progenitors through the upregulation of JmjD3 and demethylation of the tumor suppressor p16INK4a, which inhibits p53 signaling (Martinelli et al., 2011); and in B-cell lymphoma murine models, increased expressions of MAPK/ERK and Akt signaling pathways mediated by regulatory gene hypermethylation was reported (Lin et al., 2015). Altogether, these findings emphasize the possibility of developing novel prognostic and diagnostic tools for HMs based on global epigenetic changes, and the potential of epigenetic drugs for anticancer therapy improvement.

## 6 Involvement of epigenetic regulation in HMs treatment and prevention

### 6.1 Targeting epigenetic regulators for HMs therapy

The high increase in incidence of disorders and diseases like obesity, type 2 diabetes, deficit hyperactivity disorder, neurodegenerative disorders, or cancers is unlikely to be caused by changes in genetic structure of the population (Latham et al., 2012), instead it could be aggravated by the interactions between environmental risk factors and epigenetic regulation. Analyses of the human interactome, i.e., the entire set of molecular interactions in human cells, have revealed that epigenetic changes during fetal life play a pivotal role in individual disease risk, even for HMs like leukemias. Furthermore, such epigenetic changes can also play a role in the control of the expression of imprinted genes, which are a small proportion of genes whose expression occurs from only one allele inherited from parents at fertilization, such genes include IGF2 gene that is only expressed from the allele inherited from the father (Boekelheide et al., 2012). In other words, from early stages of development every person would develop an individual interactome that will determine his risk for disease development and the specific clinical features of disease. In addition, recent epidemiological and experimental data suggest the existence of a causal link between prenatal vulnerability of “future parental epigenomes” to deleterious environmental factors and the risk for developing a whole range of medical conditions, including autism, schizophrenia obesity, diabetes, asthma, and cancer (Lobanenkov et al., 2011). The effects of common environmental chemicals, such as bisphenol A and phthalates, on DNA methylome have been implicated in the etiology of diabetes and various cancers (Singh and Li, 2012). Hung and collaborators (Hung et al., 2010) reported an epigenetic regulation-mediated modulation of cytokine expression in human myeloid dendritic cells by environmental chemicals, suggesting, together with the precedent observations, that epigenetic regulation may determine the basis of biological processes resulting in the establishment of personal molecular identity, with modifications occurring throughout life.

These findings underline the importance of parameters like lifestyle and patient medical history (including drugs ever assumed) for disease treatment, considering that even common pharmaceuticals may modify the epigenome. For example, the antihypertensive hydralazine may inhibit DNA methylation, and isotretinoin (accutane), used for the treatment of severe cystic acne vulgaris, may induce stable modifications to chromatin status (Liang and Turcan, 2022). It can be anticipated that epigenetic side-effects of pharmaceuticals are involved in the development of many diseases associated with idiopathic etiology. For instance, endocrine-related cancers, in particular breast cancer, are more common in patients with a history of oral contraceptive pill use (Chandra, 2021). Moreover, significant interindividual variations have been reported in the DNA methylome of peripheral blood cells of cancer-free subjects (Zhang et al., 2011) and in leukemia cells (He et al., 2011). Major hurdles faced in attempts to translate investigational biomarkers and other experimental findings into routine clinical practice in solid cancers, such as thyroid (Gomez Saez, 2011), liver (Han, 2012), pancreatic (Ansari et al., 2012) cancers, and various HMs (Sant et al., 2010; Smith et al., 2011), are at least in part due to high interindividual variability emerging from individual “epigenetic identity”, indicating that the determination of DNA methylome status, chromatin status, and non-coding RNA changes in each patient may prove helpful for efficient anticancer therapy, particularly in drug-resistant cases. Epigenetic regulators collaborate in disease-specific, and sometimes even in individual-specific (Sant et al., 2010; Smith et al., 2011), fashions in various HMs, including AML (He et al., 2011) and B-cell lymphoma (Pasqualucci et al., 2011). Recent studies in various cancer cell lines have shown that DNA hypomethylation mechanisms interact with alkylation damage resistance to maintain genomic integrity in transforming cells in a cell-type specific manner (Dango et al., 2011), explaining at least in part the high variability of specific combinations of epigenetic changes observed at cancer type and individual levels.

### 6.2 Dietary factors and epigenetic regulation: implications for cancer control and prevention

Experimental and clinical evidence suggest that nutrients affect epigenetic regulation. A study investigating the effects of short-term high-fat overfeeding on the DNA methylome of skeletal muscle cells in healthy young men revealed a widespread change in DNA methylation affecting 6,508 genes, out of over 27,578 CpG genes investigated (Jacobsen et al., 2012). These changes were reversed slowly, suggesting that short exposure to high-fat diet sufficed to build-up methylation. Strikingly, such changes in DNA methylome affected primarily genes involved in inflammatory response, reproductive system, and cancer. Comparably, overall DNA methylation increased in liver and muscle tissues following the switch from high-fat-soy to high-fat- casein diets in *Cynomolgus* monkeys, and among others, developmental genes like homeobox genes (HOXA5, HOXA11, and HOXB1) were changed between diets (Howard et al., 2011). Furthermore, diet-induced obesity accelerated AML progression in two murine models (Yun et al., 2010). Interestingly, the methyl group supplier nutrient folic acid has been reported to have protective effects in colon cancer; folic acid deprivation resulted in an enhanced invasiveness of human colon cancer cells through promoter hypomethylation-mediated activation of the oncogenic sonic hedgehog and NF-κB signaling pathways (Wang T. P. et al., 2012). Together with the preceding, this finding suggests that diet modulations can mitigate the risk for cancer development and modify cancer clinical and pathological profile through modifications in epigenetic regulation, and consequently, should be thoroughly investigated.

Similarly, epidemiological data have suggested a protective role for physical exercise for leukemias, myelomas, and non-Hodgkin’s and Hodgkin’s lymphomas (Pan and Morrison, 2011), probably through reduction in metabolic syndrome risk factors, and in anti-inflammatory and antioxidant effects. Physical exercise modifies DNA methylation to protect against cancer risk factors like obesity and type 2 diabetes (Kabat et al., 2012; Nitert et al., 2012), and could improve patient response to anticancer classical therapies. Metabolic syndrome is commonly reported in solid cancer survivors (van et al., 2012), and several epidemiological studies have reported obesity in survivors of HMs, including childhood ALL and CLL (Quigg et al., 2012). Recent clinical studies have revealed that high levels of leptin, probably originating from epigenetic transcriptional activation (Cheng et al., 2012), are expressed in ALL survivors; during anticancer treatment, such increases are progressive and correlate with insulin resistance and increased measures of body fat (Tonorezos et al., 2012). In addition, overweight is an independent prognostic factor for relapse in ALL pediatric patients, probably due to the antiapoptotic effects in leukemia cells induced by activated adipocytes (Gelelete et al., 2011). Future studies are needed to develop strategies against morbidity associated with classical anticancer treatment in childhood cancer survivors. In particular the combination of epigenetic drugs, family-based diet, and physical exercise to classical anticancer drugs during maintenance therapy may mitigate such morbidity. A progressive body weight increase has been also reported in adult leukemia (Chu et al., 2011) and lymphoma survivors (Lynce et al., 2012) during therapy. However, epidemiological data on the impact of obesity development on therapy outcome are still controversial. Whereas some reports from the USA based studies suggest that obesity would be associated with improved overall survival in non-Hodgkin’s lymphomas (Carson et al., 2012), a report from Taiwan suggests that obesity could not be applied as a predictor of disease outcome, and central obesity would instead be used to predict non-Hodgkin’s lymphoma mortality. These differences can be explained in part by between race/ethnicity differences in DNA methylome and other epigenetic factors (Zhang et al., 2011). The combination of epigenetic drugs, diet, and physical exercise to classical anticancer drugs may also be considered in HM adult patients during maintenance therapy to mitigate obesity.

### 6.3 Targeting epigenetic regulators for cancer therapy

The first epigenetic drug approved by the United States Food and Drug Administration (FDA) was azacitidine (Vidaza^®^) in 2004 for MDS and chronic myelomonocytic leukemia (Kaminskas et al., 2005), followed by decitabine (Dacogen^®^) approved in 2006 to treat MDS (Steensma, 2009). These so-called ‘hypomethylating agents’ have been used in myeloid malignancies for more than 1 decade, even though, 50% of patients do not respond initially or during repeated cycles of treatment (Šimoničová et al., 2022). Recent studies have been reported that the DNMT1 inhibitor GSK-3484862 mediates global demethylation in murine embryonic stem cells (Šimoničová et al., 2022). Guadecitabine is a second-generation DNA methylation inhibitor being developed for AML and MDS treatment. It consists of a dinucleotide of decitabine and deoxyguanosine which is resistant to cytidine deaminase, the enzyme which is responsible for decitabine inactivation (Zhao et al., 2021).

Another class of epigenetic drugs are the histone deacetylase inhibitors (HDACi) which increase histone acetylation, an epigenetic mark of transcriptional activation, leading to an accessible chromatin conformation and promoting the expression of important genes that controls cell growth and death. As example, we have tucidinostat (chidamide), which is a novel oral subtype of selective HDACi. This drug inhibits class I HDACs (HDAC1, HDAC2, HDAC3) and class IIb (HDAC10). It was approved in 2014 as a second-line therapy for peripheral relapsed or refractory T-cell lymphoma by the China Food and Drug Administration. In Japan, tucidinostat was approved in 2021 for relapsed or refractory adult T-cell lymphoma treatment under the name Hiyasta (Sun Y. et al., 2022). Valproic acid is an FDA-approved antiepileptic drug that also presents inhibitory HDAC class I and II activity (Krauze et al., 2015). Currently, valproic acid is in phase III clinical trial as a potential drug to treat cervical and ovarian malignancies and has been proposed in combination regimens with chemotherapy and radiotherapy (Tsai et al., 2021). Compounds that modulate epigenome are being discovered and currently, there is a race in finding potential inhibitors of epigenetic modifiers. Emerging targets that modulate others DNA-modifying enzymes, as TETs and isocitrate dehydrogenase (IDHs) inhibitors (TETi and IDHi) are in current development for cancer treatment. Likewise, the complex network of histone-modifying enzymes has been added in anticancer therapy, as HMTi, HATi, HDMi, KMTi and PRMTi (Suraweera et al., 2018).

Another class of epidrugs are the inhibitors of bromodomain and extra-terminal domain (BETi), a histone “reader” that recognizes and binds to acetylated lysine and is responsible for the recruitment of transcription machinery and gene activation (Cheung et al., 2021). The combined use of epigenetic drugs with conventional therapies is gaining prominence due to its potential in increasing tumor cells’ sensitivity to classical chemotherapy improving the therapeutic effect (Fan et al., 2014; Manda et al., 2021; Sallman et al., 2021).

Epidrugs are alternative strategies for a more personalized tumor treatment because they might sensitize tumors to immune checkpoint inhibitors and cell therapy, besides their effect on viral mimicry response and immune cell activation. Currently, several clinical trials on different tumor types are ongoing using epidrugs alone or in combination with other immunotherapy drugs. There are different approaches regarding the use of epidrugs in cancer treatment, such as DNA methyltransferase inhibitors (DNMTi), histone deacetylase inhibitors HDACi, and bromodomain and extra-terminal motif inhibitors BETi.

## 7 Concluding remarks

Epigenetic regulation plays crucial roles in the maintenance of HSCs and in the development of cancer stem cells, such as LSCs. DNA methylation alterations and aberrant histone modifications are particularly relevant in the pathogenesis of both HMs and solid cancers. Epigenetic alterations resulting in DNA hypermethylation may silence tumor suppressor genes normally acting as inhibitors of oncogenic signaling pathways, such as Wnt, Notch, JAK/STAT, or Ras/MAPK pathways, whereas epigenetic deregulation resulting in DNA hypomethylation activates the genes encoding for these oncogenic signaling pathways. These Alterations result in the oncogenic pathway overexpression that contributes to the stem-like state of cancer cells. These findings suggest a key role for epigenetic regulation in the pathogenesis of HMs, and are of utmost importance for the design of new anticancer strategies, particularly for chemoresistant diseases. Differently from agents targeting only a single gene product, epigenetic drugs target the entire chromatin and gene promoter region. Hypomethylating agents like DNMTi, HDACi, and KTMi, as well as methylating agents such as KDMi and HATi, affect the whole epigenetic machinery, and may act upon most or all cancer types, as DNA methylation alterations are common hallmarks of the neoplastic transformation. Considering that epigenetic changes occur throughout lifetime in response to environmental and physiological factors, the determination of the personal “epigenetic identity” of patients may allow the design of specific epigenetic therapy particularly efficient against HMs. The use of epigenetic drugs in combination with classical anticancer drugs is proving very efficient and decreases post-treatment morbidity associated with the latter drugs, particularly in pediatric patients. Epigenetic changes in cancer, as well as drugs targeting them, should be investigated further, taking into consideration the implications for cancer biology understanding and treatment.

## Abbreviations

HMs, hematologic malignancies; BM, bone marrow.

## References

[B1] Aldhafiri F. Al-Nasser A. Al-Sugair A. Al-Mutairi H. Young D. Reilly J. J. (2012). Obesity and metabolic syndrome in adolescent survivors of standard risk childhood acute lymphoblastic leukemia in Saudi Arabia. Pediatr. Blood Cancer 59, 133–137. 10.1002/pbc.24012 22162511

[B2] Aldoss I. Capelletti M. Park J. Pistofidis R. S. Pillai R. Stiller T. (2019). Acute lymphoblastic leukemia as a clonally unrelated second primary malignancy after multiple myeloma. Leukemia 33, 266–270. 10.1038/s41375-018-0213-y 30026571 PMC9007549

[B3] Anderson O. Guttilla Reed I. K. (2020). Regulation of cell growth and migration by miR-96 and miR-183 in a breast cancer model of epithelial-mesenchymal transition. PLoS ONE 15, e0233187. 10.1371/journal.pone.0233187 32396572 PMC7217431

[B4] Ansari D. Chen B. C. Dong L. Zhou M. T. Andersson R. (2012). Pancreatic cancer: translational research aspects and clinical implications. World J. Gastroenterol. 18, 1417–1424. 10.3748/wjg.v18.i13.1417 22509073 PMC3319937

[B5] A Portilho N. Saini D. Hossain I. Sirois J. Moraes C. Pastor W. A. (2021). The DNMT1 inhibitor GSK-3484862 mediates global demethylation in murine embryonic stem cells. Epigenetics & Chromatin 14, 56. 10.1186/s13072-021-00429-0 34906184 PMC8672470

[B6] Audia J. E. Campbell R. M. (2016). Histone modifications and cancer. Cold Spring Harb. Perspect. Biol. 8, a019521. 10.1101/cshperspect.a019521 27037415 PMC4817802

[B7] Bennemann K. Galm O. Wilop S. Schubert C. Bruemmendorf T. H. Jost E. (2012). Epigenetic dysregulation of secreted frizzled-related proteins in myeloproliferative neoplasms complements the JAK2V617F-mutation. Clin. Epigenetics 4, 12. 10.1186/1868-7083-4-12 22935201 PMC3502569

[B8] Bhatla T. Wang J. Morrison D. J. Raetz E. A. Burke M. J. Brown P. (2012). Epigenetic reprogramming reverses the relapse-specific gene expression signature and restores chemosensitivity in childhood B-lymphoblastic leukemia. Blood 119, 5201–5210. 10.1182/blood-2012-01-401687 22496163 PMC3369610

[B9] Billot K. Soeur J. Chereau F. Arrouss I. Merle-Beral H. Huang M. E. (2011). Deregulation of Aiolos expression in chronic lymphocytic leukemia is associated with epigenetic modifications. Blood 117, 1917–1927. 10.1182/blood-2010-09-307140 21139082

[B10] Boekelheide K. Blumberg B. Chapin R. E. Cote I. Graziano J. H. Janesick A. (2012). Predicting later-life outcomes of early-life exposures. Environ. Health Perspect. 120, 1353–1361. 10.1289/ehp.1204934 22672778 PMC3491941

[B11] Butler J. S. Zurita-Lopez C. I. Clarke S. G. Bedford M. T. Dent S. Y. (2011). Protein-arginine methyltransferase 1 (PRMT1) methylates Ash2L, a shared component of mammalian histone H3K4 methyltransferase complexes. J. Biol. Chem. 286, 12234–12244. 10.1074/jbc.M110.202416 21285357 PMC3069427

[B12] Carson K. R. Bartlett N. L. McDonald J. R. Luo S. Zeringue A. Liu J. (2012). Increased body mass index is associated with improved survival in United States veterans with diffuse large B-cell lymphoma. J. Clin. Oncol. 30, 3217–3222. 10.1200/JCO.2011.39.2100 22649138 PMC3434980

[B13] Chandra K. (2021). Epigenetic regulation and promising therapies in colorectal cancer. Curr. Mol. Pharmacol. 14 (5), 838–852. 10.2174/1874467214666210126105345 33573584

[B14] Chen W. Liu D. Xiao H. Zhou L. Qu J. (2022). Identification of differentially expressed genes induced by aberrant methylation in acute myeloid leukemia using integrated bioinformatics analyses. Oncol. Lett. 24, 383. 10.3892/ol.2022.13503 36238356 PMC9494628

[B15] Cheng S. P. Liu C. L. Hsu Y. C. Chang Y. C. Huang S. Y. Lee J. J. (2012). Regulation of leptin receptor expression in human papillary thyroid cancer cells. Biomed. Pharmacother. 66, 469–473. 10.1016/j.biopha.2012.03.008 22560341

[B16] Cheung K. L. Kim C. Zhou M. M. (2021). The functions of BET proteins in gene transcription of biology and diseases. Front. Mol. Biosci. 8, 728777. 10.3389/fmolb.2021.728777 34540900 PMC8446420

[B17] Chu D. M. Wahlqvist M. L. Lee M. S. Chang H. Y. (2011). Central obesity predicts non-Hodgkin's lymphoma mortality and overall obesity predicts leukemia mortality in adult Taiwanese. J. Am. Coll. Nutr. 30, 310–319. 10.1080/07315724.2011.10719974 22081617

[B18] Cooper J. C. Shi M. Chueh F. Y. Venkitachalam S. Yu C. L. (2010). Enforced SOCS1 and SOCS3 expression attenuates Lck-mediated cellular transformation. Int. J. Oncol. 36, 1201–1208. 10.3892/ijo_00000603 20372794 PMC3031791

[B19] Corre J. Munshi N. C. Avet-Loiseau H. (2021). Risk factors in multiple myeloma: is it time for a revision. Blood 137, 16–19. 10.1182/blood.2019004309 33024991 PMC7808011

[B20] Dango S. Mosammaparast N. Sowa M. E. Xiong L. J. Wu F. Park K. (2011). DNA unwinding by ASCC3 helicase is coupled to ALKBH3-dependent DNA alkylation repair and cancer cell proliferation. Mol. Cell. 44, 373–384. 10.1016/j.molcel.2011.08.039 22055184 PMC3258846

[B21] Das N. D. Jung K. H. Choi M. R. Yoon H. S. Kim S. H. Chai Y. G. (2012). Gene networking and inflammatory pathway analysis in a JMJD3 knockdown human monocytic cell line. Cell. Biochem. Funct. 30, 224–232. 10.1002/cbf.1839 22252741

[B22] De B. E. Bos T. J. Schuit F. Van V. E. Menu E. Thorrez L. (2010). IGF-1 suppresses Bim expression in multiple myeloma via epigenetic and posttranslational mechanisms. Blood 115, 2430–2440. 10.1182/blood-2009-07-232801 20086250

[B23] Dorrance A. M. Yuan W. Becknell B. Arnoczky K. J. Guimond M. Strout M. P. (2006). Mll partial tandem duplication induces aberrant Hox expression *in vivo* via specific epigenetic alterations. J. Clin. Investig. 116, 2707–2716. 10.1172/JCI25546 16981007 PMC1564428

[B24] Fan H. Lu X. Wang X. Liu Y. Guo B. Zhang Y. (2014). Low-dose decitabine-based chemoimmunotherapy for patients with refractory advanced solid tumors: a phase I/II report. J. Immunol. Res. 2014, 371087. 10.1155/2014/371087 24963497 PMC4054619

[B25] Figueroa M. E. Abdel-Wahab O. Lu C. Ward P. S. Patel J. Shih A. (2010). Leukemic IDH1 and IDH2 mutations result in a hypermethylation phenotype, disrupt TET2 function, and impair hematopoietic differentiation. Cancer Cell. 18, 553–567. 10.1016/j.ccr.2010.11.015 21130701 PMC4105845

[B26] Frenzel L. P. Claus R. Plume N. Schwamb J. Konermann C. Pallasch C. P. (2011). Sustained NF-kappaB activity in chronic lymphocytic leukemia is independent of genetic and epigenetic alterations in the TNFAIP3 (A20) locus. Int. J. Cancer 128, 2495–2500. 10.1002/ijc.25579 20669229

[B27] Funakoshi-Tago M. Tsuruya R. Ueda F. Ishihara A. Kasahara T. Tamura H. (2019). Tyrosine-phosphorylated SOCS3 negatively regulates cellular transformation mediated by the myeloproliferative neoplasm-associated JAK2 V617F mutant, Cytokine 123 154753, 10.1016/j.cyto.2019.154753 31255914

[B28] Gallagher E. J. Fierz Y. Vijayakumar A. Haddad N. Yakar S. LeRoith D. (2012). Inhibiting PI3K reduces mammary tumor growth and induces hyperglycemia in a mouse model of insulin resistance and hyperinsulinemia. Oncogene 31, 3213–3222. 10.1038/onc.2011.495 22037215 PMC3275680

[B29] Geissler F. Nesic K. Kondrashova O. Dobrovic A. Swisher E. M. Scott C. L. (2024). The role of aberrant DNA methylation in cancer initiation and clinical impacts. Ther. Adv. Med. Oncol. 16, 17588359231220511. 10.1177/17588359231220511 38293277 PMC10826407

[B30] Gelelete C. B. Pereira S. H. Azevedo A. M. Thiago L. S. Mundim M. Land M. G. (2011). Overweight as a prognostic factor in children with acute lymphoblastic leukemia. Obes. (Silver Spring) 19, 1908–1911. 10.1038/oby.2011.195 21720424

[B31] Gomez Saez J. M. (2011). Diagnostic and prognostic markers in differentiated thyroid cancer. Curr. Genomics 12, 597–608. 10.2174/138920211798120826 22654559 PMC3271312

[B32] Goyama S. Nitta E. Yoshino T. Kako S. Watanabe-Okochi N. Shimabe M. (2010). EVI-1 interacts with histone methyltransferases SUV39H1 and G9a for transcriptional repression and bone marrow immortalization. Leukemia 24, 81–88. 10.1038/leu.2009.202 19776757

[B33] Greenberg P. L. Garcia-Manero G. Moore M. Damon L. Roboz G. Hu K. (2012). A randomized controlled trial of romiplostim in patients with low- or intermediate-risk myelodysplastic syndrome receiving decitabine. Leuk. Lymphoma 54, 321–328. 10.3109/10428194.2012.713477 22906162

[B34] Gruszka A. M. Valli D. Alcalay M. (2019). Wnt signalling in acute myeloid leukaemia. Cells 8, 1403. 10.3390/cells8111403 31703382 PMC6912424

[B35] Grzasko N. Hus M. Pluta A. Jurczyszyn A. Walter-Croneck A. Morawska M. (2012). Additional genetic abnormalities significantly worsen poor prognosis associated with 1q21 amplification in multiple myeloma patients. Hematol. Oncol. 31, 41–48. 10.1002/hon.2018 22674819

[B36] Guo H. Zhang T. Wen X. Zhou J. D. Ma J. An C. (2017). Hypermethylation of secreted frizzled-related proteins predicts poor prognosis in non-M3 acute myeloid leukemia. Onco Targets Ther. 10, 3635–3644. 10.2147/OTT.S136502 28790854 PMC5530859

[B37] Hackanson B. Rimmele L. Benkisser M. Abdelkarim M. Fliegauf M. Jung M. (2012). HDAC6 as a target for antileukemic drugs in acute myeloid leukemia. Leuk. Res. 36, 1055–1062. 10.1016/j.leukres.2012.02.026 22464548

[B38] Han X. Han Y. Zheng Y. Sun Q. Ma T. Zhang J. (2017). Chaetocin induces apoptosis in human melanoma cells through the generation of reactive oxygen species and the intrinsic mitochondrial pathway, and exerts its anti-tumor activity *in vivo* . PLoS ONE 12, e0175950. 10.1371/journal.pone.0175950 28419143 PMC5395229

[B39] Han Z. G. (2012). Functional genomic studies: insights into the pathogenesis of liver cancer. Annu. Rev. Genomics Hum. Genet. 13, 171–205. 10.1146/annurev-genom-090711-163752 22703171

[B40] He J. Nguyen A. T. Zhang Y. (2011). KDM2b/JHDM1b, an H3K36me2-specific demethylase, is required for initiation and maintenance of acute myeloid leukemia. Blood 117, 3869–3880. 10.1182/blood-2010-10-312736 21310926 PMC3083299

[B41] He S. Zhou J. Yu L. (2022). Aberrant DNA methylation in t(8;21) acute myeloid leukemia. DIS. 3, 209–216. 10.1007/s42764-022-00074-1

[B42] Hogan L. E. Meyer J. A. Yang J. Wang J. Wong N. Yang W. (2011). Integrated genomic analysis of relapsed childhood acute lymphoblastic leukemia reveals therapeutic strategies. Blood 118, 5218–5226. 10.1182/blood-2011-04-345595 21921043 PMC3217405

[B43] Hopfer O. Nolte F. Mossner M. Komor M. Kmetsch A. Benslasfer O. (2012). Epigenetic dysregulation of GATA1 is involved in myelodysplastic syndromes dyserythropoiesis. Eur. J. Haematol. 88, 144–153. 10.1111/j.1600-0609.2011.01715.x 21967505

[B44] Hou H. A. Kuo Y. Y. Liu C. Y. Chou W. C. Lee M. C. Chen C. Y. (2012). DNMT3A mutations in acute myeloid leukemia: stability during disease evolution and clinical implications. Blood 119, 559–568. 10.1182/blood-2011-07-369934 22077061

[B45] Howard T. D. Ho S. M. Zhang L. Chen J. Cui W. Slager R. (2011). Epigenetic changes with dietary soy in cynomolgus monkeys. PLoS One 6, e26791. 10.1371/journal.pone.0026791 22046358 PMC3201974

[B46] Hua W. F. Fu Y. S. Liao Y. J. Xia W. J. Chen Y. C. Zeng Y. X. (2010). Curcumin induces down-regulation of EZH2 expression through the MAPK pathway in MDA-MB-435 human breast cancer cells. Eur. J. Pharmacol. 637, 16–21. 10.1016/j.ejphar.2010.03.051 20385124

[B47] Hung C. H. Yang S. N. Kuo P. L. Chu Y. T. Chang H. W. Wei W. J. (2010). Modulation of cytokine expression in human myeloid dendritic cells by environmental endocrine-disrupting chemicals involves epigenetic regulation. Environ. Health Perspect. 118, 67–72. 10.1289/ehp.0901011 20056579 PMC2831970

[B48] Ismail N. H. Mussa A. Al-Khreisat M. J. Mohamed Yusoff S. Husin A. Al-Jamal H. A. N. (2023). Dysregulation of non-coding RNAs: roles of miRNAs and lncRNAs in the pathogenesis of multiple myeloma. Non-Coding RNA 9, 68. 10.3390/ncrna9060068 37987364 PMC10660696

[B49] Ismail N. H. Mussa A. Zakaria N. A. Al-Khreisat M. J. Zahidin M. A. Ramli N. N. (2022). The role of epigenetics in the development and progression of multiple myeloma. Biomedicines 10, 2767. 10.3390/biomedicines10112767 36359286 PMC9687797

[B50] Issah M. A. Wu D. Zhang F. Zheng W. Liu Y. Fu H. (2021). Epigenetic modifications in acute myeloid leukemia: the emerging role of circular RNAs (Review). Int. J. Oncol. 59, 107. 10.3892/ijo.2021.5287 34792180 PMC8651224

[B51] Jacobsen S. C. Brons C. Bork-Jensen J. Ribel-Madsen R. Yang B. Lara E. (2012). Effects of short-term high-fat overfeeding on genome-wide DNA methylation in the skeletal muscle of healthy young men. Diabetologia 55, 3341–3349. 10.1007/s00125-012-2717-8 22961225

[B52] Jeannet R. Mastio J. Macias-Garcia A. Oravecz A. Ashworth T. Geimer Le Lay A. S. (2010). Oncogenic activation of the Notch1 gene by deletion of its promoter in Ikaros-deficient T-ALL. Blood 116, 5443–5454. 10.1182/blood-2010-05-286658 20829372 PMC3100247

[B53] Jenke R. Reßing N. Hansen F. K. Aigner A. Büch T. (2021). Anticancer therapy with HDAC inhibitors: mechanism-based combination strategies and future perspectives. Cancers (Basel) 13, 634. 10.3390/cancers13040634 33562653 PMC7915831

[B54] Jeusset L. M. McManus K. J. (2019). Developing targeted therapies that exploit aberrant histone ubiquitination in cancer. Cells 8, 165. 10.3390/cells8020165 30781493 PMC6406838

[B55] Kabat G. C. Kim M. Y. Jean W. W. Bea J. W. Edlefsen K. L. Adams-Campbell L. L. (2012). Anthropometric factors, physical activity, and risk of non-Hodgkin's lymphoma in the Women's Health Initiative. Cancer Epidemiol. 36, 52–59. 10.1016/j.canep.2011.05.014 21816698 PMC6086363

[B56] Kalinkova L. Sevcikova A. Stevurkova V. Fridrichova I. Ciernikova S. (2023). Targeting DNA methylation in leukemia, myelodysplastic syndrome, and lymphoma: a potential diagnostic, prognostic, and therapeutic tool. Int. J. Mol. Sci., 24, 633. 10.3390/ijms24010633 PMC9820560 36614080

[B57] Kaminskas E. Farrell A. Abraham S. Baird A. Hsieh L. S. Lee S. L. (2005). Approval summary: azacitidine for treatment of myelodysplastic syndrome subtypes. Clin. Cancer Res. 11, 3604–3608. 10.1158/1078-0432.CCR-04-2135 15897554

[B58] Kantarjian H. M. Thomas X. G. Dmoszynska A. Wierzbowska A. Mazur G. Mayer J. (2012). Multicenter, randomized, open-label, phase III trial of decitabine versus patient choice, with physician advice, of either supportive care or low-dose cytarabine for the treatment of older patients with newly diagnosed acute myeloid leukemia. J. Clin. Oncol. 30, 2670–2677. 10.1200/jco.2011.38.9429 22689805 PMC4874148

[B59] Karakurt H. Sarper N. Kilic S. C. Gelen S. A. Zengin E. (2012). Screening survivors of childhood acute lymphoblastic leukemia for obesity, metabolic syndrome, and insulin resistance. Pediatr. Hematol. Oncol. 29, 551–561. 10.3109/08880018.2012.708892 22897748

[B60] Khandanpour C. Krongold J. Schutte J. Bouwman F. Vassen L. Gaudreau M. C. (2012). The human GFI136N variant induces epigenetic changes at the Hoxa9 locus and accelerates K-RAS driven myeloproliferative disorder in mice. Blood 120, 4006–4017. 10.1182/blood-2011-02-334722 22932805

[B61] Kikuchi H. Kuribayashi F. Takami Y. Imajoh-Ohmi S. Nakayama T. (2011). GCN5 regulates the activation of PI3K/Akt survival pathway in B cells exposed to oxidative stress via controlling gene expressions of Syk and Btk. Biochem. Biophys. Res. Commun. 405, 657–661. 10.1016/j.bbrc.2011.01.088 21281601

[B62] Kim J. Y. Kim K. B. Eom G. H. Choe N. Kee H. J. Son H. J. (2012a). KDM3B is the H3K9 demethylase involved in transcriptional activation of lmo2 in leukemia. Mol. Cell. Biol. 32, 2917–2933. 10.1128/MCB.00133-12 22615488 PMC3416203

[B63] Kim S. W. Ramasamy K. Bouamar H. Lin A. P. Jiang D. Aguiar R. C. (2012b). MicroRNAs miR-125a and miR-125b constitutively activate the NF-κB pathway by targeting the tumor necrosis factor alpha-induced protein 3 (TNFAIP3, A20). Proc. Natl. Acad. Sci. U. S. A. 109, 7865–7870. 10.1073/pnas.1200081109 22550173 PMC3356650

[B64] Kimbrel E. A. Lemieux M. E. Xia X. Davis T. N. Rebel V. I. Kung A. L. (2009). Systematic *in vivo* structure-function analysis of p300 in hematopoiesis. Blood 114, 4804–4812. 10.1182/blood-2009-04-217794 19822904

[B65] Krauze A. V. Myrehaug S. D. Chang M. G. Holdford D. J. Smith S. Shih J. (2015). A phase 2 study of concurrent radiation therapy, temozolomide, and the histone deacetylase inhibitor valproic acid for patients with glioblastoma. Int. J. Radiat. Oncol. Biol. Phys. 92, 986–992. 10.1016/j.ijrobp.2015.04.038 26194676 PMC4510472

[B66] Kuang S. Q. Bai H. Fang Z. H. Lopez G. Yang H. Tong W. (2010). Aberrant DNA methylation and epigenetic inactivation of Eph receptor tyrosine kinases and ephrin ligands in acute lymphoblastic leukemia. Blood 115, 2412–2419. 10.1182/blood-2009-05-222208 20061560 PMC2845899

[B67] Lafave L. M. Levine R. L. (2012). JAK2 the future: therapeutic strategies for JAK-dependent malignancies. Trends Pharmacol. Sci. 33, 574–582. 10.1016/j.tips.2012.08.005 22995223

[B68] Latham K. E. Sapienza C. Engel N. (2012). The epigenetic lorax: gene-environment interactions in human health. Epigenomics 4, 383–402. 10.2217/epi.12.31 22920179 PMC3471221

[B69] Lau A. T. Lee S. Y. Xu Y. M. Zheng D. Cho Y. Y. Zhu F. (2011). Phosphorylation of histone H2B serine 32 is linked to cell transformation. J. Biol. Chem. 286, 26628–26637. 10.1074/jbc.M110.215590 21646345 PMC3143627

[B70] Liang Y. Turcan S. (2022). Epigenetic drugs and their immune modulating potential in cancers. Biomedicines 10, 211. 10.3390/biomedicines10020211 35203421 PMC8868629

[B71] Liddiard K. Hills R. Burnett A. K. Darley R. L. Tonks A. (2010). OGG1 is a novel prognostic indicator in acute myeloid leukaemia. Oncogene 29, 2005–2012. 10.1038/onc.2009.462 20023702

[B72] Lin C. Chiang W. Tung C. Hsieh J. L. Hsiao J. Huang W. (2015). Sprouty2 protein is downregulated in human squamous cell carcinoma of the head and neck and suppresses cell proliferation *in vitro* . Mol. Med. Rep. 11, 547–554. 10.3892/mmr.2014.2700 25333206

[B73] Liu F. Karube K. Kato H. Arita K. Yoshida N. Yamamoto K. (2012). Mutation analysis of NF-κB signal pathway-related genes in ocular MALT lymphoma. Int. J. Clin. Exp. Pathol. 5, 436–441. 22808296 PMC3396059

[B74] Liu X. L. Liu H. Q. Li J. Mao C. Y. He J. T. Zhao X. (2020). Role of epigenetic in leukemia: from mechanism to therapy. Chem. Biol. Interact. 317, 108963. 10.1016/j.cbi.2020.108963 31978391

[B75] Lobanenkov V. Loukinov D. Pugacheva E. ((2011)). Environmental epigenomics and disease susceptibility. Keystone symposia on molecular and cellular biology. Grove Park Hotel & Spa, Ashv. 3 261–266. 10.2217/epi.11.25 22122336

[B76] Lynce F. Pehlivanova M. Catlett J. Malkovska V. (2012). Obesity in adult lymphoma survivors. Leuk. Lymphoma 53, 569–574. 10.3109/10428194.2011.619606 21888618

[B77] Ma W. Gutierrez A. Goff D. J. Geron I. Sadarangani A. Jamieson C. A. (2012). NOTCH1 signaling promotes human T-cell acute lymphoblastic leukemia initiating cell regeneration in supportive niches. PLoS One 7, e39725. 10.1371/journal.pone.0039725 22768113 PMC3387267

[B78] Ma Y. Wang S. Yang M. Bao J. Wang C. (2020). Analysis of risk factors and clinical indicators in bloodstream infections among patients with hematological malignancy. Cancer Manag. Res. 12, 13579–13588. 10.2147/CMAR.S289291 33408527 PMC7780855

[B79] Manda S. Anz B. Benton C. Broun E. R. Yimer H. Renshaw J. (2021). Treatment initiation of venetoclax in combination with azacitidine or decitabine in an outpatient setting in patients with untreated acute myeloid leukemia. Blood 138, 1265. 10.1182/blood-2021-144886

[B80] Markopoulos G. S. Roupakia E. Tokamani M. Alabasi G. Sandaltzopoulos R. Marcu K. B. (2018). Roles of NF-κB signaling in the regulation of miRNAs impacting on inflammation in cancer. Biomedicines 6, 40. 10.3390/biomedicines6020040 29601548 PMC6027290

[B81] Martinelli P. Bonetti P. Sironi C. Pruneri G. Fumagalli C. Raviele P. R. (2011). The lymphoma-associated NPM-ALK oncogene elicits a p16INK4a/pRb-dependent tumor-suppressive pathway. Blood 117, 6617–6626. 10.1182/blood-2010-08-301135 21518927

[B82] Melo-Cardenas J. Xu Y. Wei J. Tan C. Kong S. Gao B. (2018). USP22 deficiency leads to myeloid leukemia upon oncogenic Kras activation through a PU.1-dependent mechanism. Blood 132, 423–434. 10.1182/blood-2017-10-811760 29844011 PMC6071563

[B83] Metzger E. Imhof A. Patel D. Kahl P. Hoffmeyer K. Friedrichs N. (2010). Phosphorylation of histone H3T6 by PKCbeta(I) controls demethylation at histone H3K4. Nature 464, 792–796. 10.1038/nature08839 20228790

[B84] Moskalev E. A. Luckert K. Vorobjev I. A. Mastitsky S. E. Gladkikh A. A. Stephan A. (2012). Concurrent epigenetic silencing of wnt/β-catenin pathway inhibitor genes in B cell chronic lymphocytic leukaemia. BMC Cancer 12, 213. 10.1186/1471-2407-12-213 22672427 PMC3489542

[B85] Mullighan C. G. Zhang J. Kasper L. H. Lerach S. Payne-Turner D. Phillips L. A. (2011). CREBBP mutations in relapsed acute lymphoblastic leukaemia. Nature 471, 235–239. 10.1038/nature09727 21390130 PMC3076610

[B86] Nakamura S. Oshima M. Yuan J. Saraya A. Miyagi S. Konuma T. (2012). Bmi1 confers resistance to oxidative stress on hematopoietic stem cells. PLoS One 7, e36209. 10.1371/journal.pone.0036209 22606246 PMC3350495

[B87] Newton K. Dixit V. M. (2012). Signaling in innate immunity and inflammation. Cold Spring Harb. Perspect. Biol. 4, a006049. 10.1101/cshperspect.a006049 22296764 PMC3282411

[B88] Nishioka C. Ikezoe T. Yang J. Yokoyama A. (2010). Long-term exposure of leukemia cells to multi-targeted tyrosine kinase inhibitor induces activations of AKT, ERK and STAT5 signaling via epigenetic silencing of the PTEN gene. Leukemia 24, 1631–1640. 10.1038/leu.2010.145 20596030

[B89] Nitert M. D. Dayeh T. Volkov P. Elgzyri T. Hall E. Nilsson E. (2012). Impact of an exercise intervention on DNA methylation in skeletal muscle from first-degree relatives of patients with type 2 diabetes. Diabetes 61, 3322–3332. 10.2337/db11-1653 23028138 PMC3501844

[B90] Oliveira L. P. de Jesus R. P. Boulhosa R. S. Mendes C. M. Lyra A. C. Lyra L. G. (2012). Metabolic syndrome in patients with chronic hepatitis C virus genotype 1 infection who do not have obesity or type 2 diabetes. Clin. (Sao Paulo) 67, 219–223. 10.6061/clinics/2012(03)03 PMC3297029 22473401

[B91] Pan S. Y. Morrison H. (2011). Physical activity and hematologic cancer prevention. Cancer Res. 186, 135–158. 10.1007/978-3-642-04231-7_6 21113763

[B92] Pasqualucci L. Dominguez-Sola D. Chiarenza A. Fabbri G. Grunn A. Trifonov V. (2011). Inactivating mutations of acetyltransferase genes in B-cell lymphoma. Nature 471, 189–195. 10.1038/nature09730 21390126 PMC3271441

[B93] Peng L. Seto E. (2011). Deacetylation of nonhistone proteins by HDACs and the implications in cancer. Handb. Exp. Pharmacol. 206, 39–56. 10.1007/978-3-642-21631-2_3 21879445

[B94] Qin J. Wen B. Liang Y. Yu W. Li H. (2020). Histone modifications and their role in colorectal cancer (review). Pathol. Oncol. Res. 26, 2023–2033. 10.1007/s12253-019-00663-8 31055775 PMC7471167

[B95] Quagliano A. Gopalakrishnapillai A. Barwe S. P. (2020). Understanding the mechanisms by which epigenetic modifiers avert therapy resistance in cancer. Front. Oncol. 10, 992. 10.3389/fonc.2020.00992 32670880 PMC7326773

[B96] Quigg T. C. Haddad N. G. Buchsbaum J. C. Shih C. S. (2012). Hypothalamic obesity syndrome: rare presentation of CNS+ B-cell lymphoblastic lymphoma. Pediatr. Blood Cancer 59, 930–933. 10.1002/pbc.24058 22213612 PMC4020003

[B97] Reins J. Mossner M. Neumann M. Platzbecker U. Schumann C. Thiel E. (2010). Transcriptional down-regulation of the Wnt antagonist SFRP1 in haematopoietic cells of patients with different risk types of MDS. Leuk. Res. 34, 1610–1616. 10.1016/j.leukres.2010.04.013 20471677

[B98] Rosenberg A. S. (2022). From mechanism to resistance - changes in the use of dexamethasone in the treatment of multiple myeloma. Leuk. Lymphoma 28, 283–291. 10.1080/10428194.2022.2136950 36308022

[B99] Roy I. Ghosh G. (2021). Aberrant signaling pathways in cancer cells: application of nanomaterials. J. Cell. Signal 2, 281–299.

[B100] Sallman D. A. DeZern A. E. Garcia-Manero G. Steensma D. P. Roboz G. J. Sekeres M. A. (2021). Eprenetapopt (APR-246) and azacitidine in TP53-mutant myelodysplastic syndromes. J. Clin. Oncol. 39, 1584–1594. 10.1200/JCO.20.02341 33449813 PMC8099410

[B101] Sant M. Allemani C. Tereanu C. De A. R. Capocaccia R. Visser O. (2010). Incidence of hematologic malignancies in Europe by morphologic subtype: results of the HAEMACARE project. Blood 116, 3724–3734. 10.1182/blood-2010-05-282632 20664057

[B102] Shankar S. Srivastava R. K. (2008). Histone deacetylase inhibitors: mechanisms and clinical significance in cancer: HDAC inhibitor-induced apoptosis. Adv. Exp. Med. Biol. 615, 261–298. 10.1007/978-1-4020-6554-5_13 18437899

[B103] Shanmugam M. K. Arfuso F. Arumugam S. Chinnathambi A. Jinsong B. Warrier S. (2017). Role of novel histone modifications in cancer. Oncotarget 9, 11414–11426. 10.18632/oncotarget.23356 29541423 PMC5834259

[B104] Shanmugam R. Gade P. Wilson-Weekes A. Sayar H. Suvannasankha A. Goswami C. (2012). A noncanonical Flt3ITD/NF-κB signaling pathway represses DAPK1 in acute myeloid leukemia. Clin. Cancer Res. 18, 360–369. 10.1158/1078-0432.CCR-10-3022 22096027 PMC3918433

[B105] Šimoničová K. Janotka L. Kavcová H. Sulová Z. Breier A. Messingerova L. (2022). Different mechanisms of drug resistance to hypomethylating agents in the treatment of myelodysplastic syndromes and acute myeloid leukemia. Drug resist. Updat 61, 100805. 10.1016/j.drup.2022.100805 35227933

[B106] Singh S. Li S. S. (2012). Epigenetic effects of environmental chemicals bisphenol a and phthalates. Int. J. Mol. Sci. 13, 10143–10153. 10.3390/ijms130810143 22949852 PMC3431850

[B107] Smith A. Howell D. Patmore R. Jack A. Roman E. (2011). Incidence of haematological malignancy by sub-type: a report from the haematological malignancy research network. Br. J. Cancer 105, 1684–1692. 10.1038/bjc.2011.450 22045184 PMC3242607

[B108] Son H. J. Kim J. Y. Hahn Y. Seo S. B. (2012). Negative regulation of JAK2 by H3K9 methyltransferase G9a in leukemia. Mol. Cell. Biol. 32, 3681–3694. 10.1128/MCB.00673-12 22801367 PMC3430193

[B109] Stanczyk M. Sliwinski T. Cuchra M. Zubowska M. Bielecka-Kowalska A. Kowalski M. (2011). The association of polymorphisms in DNA base excision repair genes XRCC1, OGG1 and MUTYH with the risk of childhood acute lymphoblastic leukemia. Mol. Biol. Rep. 38, 445–451. 10.1007/s11033-010-0127-x 20364408

[B110] Steensma D. P. (2009). Decitabine treatment of patients with higher-risk myelodysplastic syndromes. Leuk. Res. 33, S12–S17. 10.1016/S0145-2126(09)70228-0 20004791

[B111] Sun S. Yu F. Xu D. Zheng H. Li M. (2022a). EZH2, a prominent orchestrator of genetic and epigenetic regulation of solid tumor microenvironment and immunotherapy. Biochimica Biophysica Acta (BBA) - Rev. Cancer 1877, 188700. 10.1016/j.bbcan.2022.188700 35217116

[B112] Sun Y. Hong J. H. Ning Z. Pan D. Fu X. Lu X. (2022b). Therapeutic potential of tucidinostat, a subtype-selective HDAC inhibitor, in cancer treatment. Front. Pharmacol. 13, 932914. 10.3389/fphar.2022.932914 36120308 PMC9481063

[B113] Suraweera A. O’Byrne K. J. Richard D. J. (2018). Combination therapy with histone deacetylase inhibitors (HDACi) for the treatment of cancer: achieving the full therapeutic potential of HDACi. Front. Oncol. 8, 92. 10.3389/fonc.2018.00092 29651407 PMC5884928

[B114] Takao S. Forbes L. Uni M. Cheng S. Pineda J. M. B. Tarumoto Y. (2021). Convergent organization of aberrant MYB complex controls oncogenic gene expression in acute myeloid leukemia. eLife 10, e65905. 10.7554/eLife.65905 33527899 PMC7886351

[B115] Tonorezos E. S. Vega G. L. Sklar C. A. Chou J. F. Moskowitz C. S. Mo Q. (2012). Adipokines, body fatness, and insulin resistance among survivors of childhood leukemia. Pediatr. Blood Cancer 58, 31–36. 10.1002/pbc.22964 21254377 PMC3520427

[B116] Trowbridge J. J. Sinha A. U. Zhu N. Li M. Armstrong S. A. Orkin S. H. (2012). Haploinsufficiency of Dnmt1 impairs leukemia stem cell function through derepression of bivalent chromatin domains. Genes. Dev. 26, 344–349. 10.1101/gad.184341.111 22345515 PMC3289882

[B117] Tsai H. C. Wei K. C. Chen P. Y. Huang C. Y. Chen K. T. Lin Y. J. (2021). Valproic acid enhanced temozolomide-induced anticancer activity in human glioma through the p53–PUMA apoptosis pathway. Front. Oncol. 11, 722754. 10.3389/fonc.2021.722754 34660288 PMC8518553

[B118] Tzivion G. Dobson M. Ramakrishnan G. (2011). FoxO transcription factors; Regulation by AKT and 14-3-3 proteins. Biochim. Biophys. Acta 1813, 1938–1945. 10.1016/j.bbamcr.2011.06.002 21708191

[B119] Urbano A. Smith J. Weeks R. J. Chatterjee A. (2019). Gene-specific targeting of DNA methylation in the mammalian genome. Cancers Basel 11, 1515. 10.3390/cancers11101515 31600992 PMC6827012

[B120] van W. M. Neggers S. J. Uitterlinden A. G. Blijdorp K. van der Geest I. M. Pieters R. (2012). Treatment factors rather than genetic variation determine metabolic syndrome in childhood cancer survivors. Eur. J. Cancer. 10.1016/j.ejca.2012.09.007 23036851

[B121] Veneti Z. Gkouskou K. K. Eliopoulos A. G. (2017). Polycomb repressor complex 2 in genomic *Instability and cancer* int J mol sci. Int. J. Mol. Sci. 18, 1657. 10.3390/ijms18081657 28758948 PMC5578047

[B122] Verras M. Papandreou I. Denko N. C. (2015). WNT16-expressing acute lymphoblastic leukemia cells are sensitive to autophagy inhibitors after ER stress induction. Anticancer Res. 35, 4625–4631. 26254351 PMC4810683

[B123] Vrábel D. Pour L. Ševčíková S. (2019). The impact of NF-κB signaling on pathogenesis and current treatment strategies in multiple myeloma. Blood Rev. 34, 56–66. 10.1016/j.blre.2018.11.003 30501907

[B124] Wallace L. Obeng E. A. (2023). Noncoding rules of survival: epigenetic regulation of normal and malignant hematopoiesis. Front. Mol. Biosci. 10, 1273046. 10.3389/fmolb.2023.1273046 38028538 PMC10644717

[B125] Wallin A. Larsson S. C. (2011). Body mass index and risk of multiple myeloma: a meta-analysis of prospective studies. Eur. J. Cancer 47, 1606–1615. 10.1016/j.ejca.2011.01.020 21354783

[B126] Wang H. Xu J. Lazarovici P. Zheng R. Q. W. (2018). cAMP response element-binding protein (CREB): a possible signaling molecule link in the pathophysiology of schizophrenia. Front. Mol. Neurosci. 11, 255. 10.3389/fnmol.2018.00255 30214393 PMC6125665

[B127] Wang H. C. Peng V. Zhao Y. Sun X. H. (2012b). Enhanced Notch activation is advantageous but not essential for T cell lymphomagenesis in Id1 transgenic mice. PLoS One 7, e32944. 10.1371/journal.pone.0032944 22393458 PMC3290631

[B128] Wang J. C. Kafeel M. I. Avezbakiyev B. Chen C. Sun Y. Rathnasabapathy C. (2011). Histone deacetylase in chronic lymphocytic leukemia. Oncology 81, 325–329. 10.1159/000334577 22237050

[B129] Wang T. P. Hsu S. H. Feng H. C. Huang R. F. (2012a). Folate deprivation enhances invasiveness of human colon cancer cells mediated by activation of sonic hedgehog signaling through promoter hypomethylation and cross action with transcription nuclear factor-kappa B pathway. Carcinogenesis 33, 1158–1168. 10.1093/carcin/bgs138 22461522

[B130] Weng M. S. Chang J. H. Hung W. Y. Yang Y.-C. Hchien M. (2018). The interplay of reactive oxygen species and the epidermal growth factor receptor in tumor progression and drug resistance. J. Exp. Clin. Cancer Res. 37, 61. 10.1186/s13046-018-0728-0 29548337 PMC5857086

[B131] Wolf C. Garding A. Filarsky K. Bahlo J. Robrecht S. Becker N. (2018). NFATC1 activation by DNA hypomethylation in chronic lymphocytic leukemia correlates with clinical staging and can be inhibited by ibrutinib. Int. J. Cancer 142, 322–333. 10.1002/ijc.31057 28921505

[B132] Wolyniec K. Shortt J. de S. E. Levav-Cohen Y. Alsheich-Bartok O. Louria-Hayon I. (2012). E6AP ubiquitin ligase regulates PML-induced senescence in Myc-driven lymphomagenesis. Blood 120, 822–832. 10.1182/blood-2011-10-387647 22689861 PMC3709628

[B133] Woo A. J. Kim J. Xu J. Huang H. Cantor A. B. (2011). Role of ZBP-89 in human globin gene regulation and erythroid differentiation. Blood 118, 3684–3693. 10.1182/blood-2011-03-341446 21828133 PMC3186340

[B134] Wu S. Zhu W. Thompson P. Hannun Y. A. (2018). Evaluating intrinsic and non-intrinsic cancer risk factors. Nat. Commun. 9, 3490. 10.1038/s41467-018-05467-z 30154431 PMC6113228

[B135] Xie Z. Chng W. J. (2014). MMSET: role and therapeutic opportunities in multiple myeloma. Biomed. Res. Int. 2014, 636514. 10.1155/2014/636514 25093175 PMC4100374

[B136] Yamagishi M. Nakano K. Miyake A. Yamochi T. Kagami Y. Tsutsumi A. (2012). Polycomb-mediated loss of miR-31 activates NIK-dependent NF-κB pathway in adult T cell leukemia and other cancers. Cancer Cell. 21, 121–135. 10.1016/j.ccr.2011.12.015 22264793

[B137] Yang X. Wong M. P. M. Ng R. K. (2019). Aberrant DNA methylation in acute myeloid leukemia and its clinical implications. Int. J. Mol. Sci. 20, 4576. 10.3390/ijms20184576 31527484 PMC6770227

[B138] Yang X.-J. (2015). MOZ and MORF acetyltransferases: molecular interaction, animal development and human disease. BBA - Mol. Cell. Res. 1853, 1818–1826. 10.1016/j.bbamcr.2015.04.014 25920810

[B139] Yatim A. Benne C. Sobhian B. Laurent-Chabalier S. Deas O. Judde J. G. (2012). NOTCH1 nuclear interactome reveals key regulators of its transcriptional activity and oncogenic function. Mol. Cell. 48, 445–458. 10.1016/j.molcel.2012.08.022 23022380 PMC3595990

[B140] Yoshimi A. Goyama S. Watanabe-Okochi N. Yoshiki Y. Nannya Y. Nitta E. (2011). Evi1 represses PTEN expression and activates PI3K/AKT/mTOR via interactions with polycomb proteins. Blood 117, 3617–3628. 10.1182/blood-2009-12-261602 21289308

[B141] Yun J. P. Behan J. W. Heisterkamp N. Butturini A. Klemm L. Ji L. (2010). Diet-induced obesity accelerates acute lymphoblastic leukemia progression in two murine models. Cancer Prev. Res. (Phila) 3, 1259–1264. 10.1158/1940-6207.CAPR-10-0087 20823291 PMC2955776

[B142] Zhang F. F. Cardarelli R. Carroll J. Fulda K. G. Kaur M. Gonzalez K. (2011). Significant differences in global genomic DNA methylation by gender and race/ethnicity in peripheral blood. Epigenetics 6, 623–629. 10.4161/epi.6.5.15335 21739720 PMC3230547

[B143] Zhang H. He J. Li J. Tian D. Gu L. Zhou M. (2012). Methylation of RASSF1A gene promoter is regulated by p53 and DAXX. FASEB J. 27, 232–242. 10.1096/fj.12-215491 23038753 PMC3528318

[B144] Zhang J. Gao X. Yu L. (2021). Roles of histone deacetylases in acute myeloid leukemia with fusion proteins. Front. Oncol. 11, 741746. 10.3389/fonc.2021.741746 34540702 PMC8440836

[B145] Zhang L. Zhou L. Shi M. Kuang Y. Fang L. (2018). Downregulation of miRNA-15a and miRNA-16 promote tumor proliferation in multiple myeloma by increasing CABIN1 expression. Oncol. Lett. 15, 1287–1296. 10.3892/ol.2017.7424 29399181 PMC5772676

[B146] Zhang P. Zhang M. (2020). Epigenetic alterations and advancement of treatment in peripheral T-cell lymphoma. Clin. Epigenetics 12 (1), 169. 10.1186/s13148-020-00962-x 33160401 PMC7648940

[B147] Zhao G. Wang Q. Li S. Wang X. (2021). Resistance to hypomethylating agents in myelodysplastic syndrome and acute myeloid leukemia from clinical data and molecular mechanism. Front. Oncol. 11, 706030. 10.3389/fonc.2021.706030 34650913 PMC8505973

[B148] Zhong J. (2016). RAS and downstream RAF-MEK and PI3K-AKT signaling in neuronal development, function and dysfunction. Biol. Chem. 397, 215–222. 10.1515/hsz-2015-0270 26760308 PMC4753800

